# p21‐Activated Kinase 1 (Pak1) as an Element in Functional and Dysfunctional Interplay Among the Myocardium, Adipose Tissue, and Pancreatic Beta Cells

**DOI:** 10.1002/cph4.70006

**Published:** 2025-03-10

**Authors:** Paola C. Rosas, R. John Solaro

**Affiliations:** ^1^ Department of Pharmacy Practice, College of Pharmacy University of Illinois at Chicago Chicago Illinois USA; ^2^ Department of Physiology and Biophysics, College of Medicine University of Illinois at Chicago Chicago Illinois USA

**Keywords:** adipokines, arrythmias, cardiokines, heart failure, Hippo signaling, obesity

## Abstract

This review focuses on p21‐activated kinase 1 (Pak1), a multifunctional, highly conserved enzyme that regulates multiple downstream effectors present in many tissues. Upstream signaling via Ras‐related small G‐proteins, Cdc42/Rac1 promotes the activity of Pak1. Our hypothesis is that this signaling cascade is an important element in communication among the myocardium, adipose tissue, and pancreatic β‐cells. Evidence indicates that a shared property of these tissues is that structure/function stability requires homeostatic Pak1 activity. Increases or decreases in Pak1 activity may promote dysfunction or increase susceptibility to stressors. Evidence that increased levels of Pak1 activity may be protective provides support for efforts to develop therapeutic approaches activating Pak1 with potential use in prevalent disorders associated with obesity, diabetes, and myocardial dysfunction. On the other hand, since increased Pak1 activity is associated with cancer progression, there has been a significant effort to develop Pak1 inhibitors. These opposing therapeutic approaches highlight the need for a deep understanding of Pak1 signaling in relation to the development of effective and selective therapies with minimal or absent off‐target effects.

## Graphical abstract

Pak1 regulates cardiac function, adipose tissue metabolism, and pancreatic β‐cell survival and function. Inflammation, oxidative stress, and Hippo signaling link Pak1 dysfunction across these tissues. Pak1 activators hold promise as potential therapeutic interventions. Images were created with a licensed version of BioRender.com.

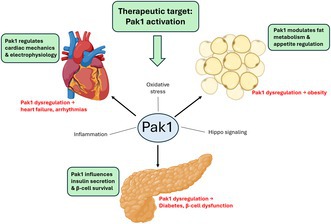

## 1 Introduction

Balanced reciprocal communication among the myocardium, adipose tissue, and pancreatic β‐cells is a crucial and intricate homeostatic process. This homeostatic interplay provides stability of function in the face of physiological stresses such as exercise. Apart from providing insulin signaling to both adipose tissue and the heart, substrates for energy metabolism by the mobilization of free fatty acids from fat depots to the heart, there are overlapping signals among these elements that ensure whole‐body metabolic, functional, and mechanical/electrical stability. There is also an abundance of evidence indicating that the homeostatic function of the insulin‐secreting β‐cells is required for the stabilization of myocardial function and adipose tissue.

Importantly, there are worldwide health effects associated with an out‐of‐balance communication among adipose tissue, pancreatic β‐cells, and the heart. The pathological interplay between adipocyte dysfunction in obesity and diabetes and the failure of cardiac function provides a vivid example of adverse effects when this communication is out of balance. Estimates indicate that among Americans over 20 years old, around 6.7 million have heart failure (HF) with a prediction that the prevalence will grow to 8.7 million in 2030, 10.3 million in 2040, and 11.4 million by 2050 (Bozkurt et al. 2025). Obesity, which is considered a global epidemic with over half of the world's population and well over half of the American population considered overweight, doubles the prevalence of HF with preserved ejection fraction (HFpEF) (Haass et al. 2011). Studies by Kenchaiah et al. (2002) concluded that 11% of HF cases in men and 14% in women are due mainly to obesity alone. Moreover, therapies that are effective for HFrEF (heart failure with reduced ejection fraction) have not demonstrated the same effectiveness in HFpEF, leaving HFpEF with only a limited selection of effective drugs (Anker et al. 2021; Basile et al. 2023; Pfeffer et al. 2015; Solomon et al. 2022). Thus, an unmet need persists for detailed understanding of the mechanisms of crosstalk between adipose tissue, pancreatic β cells, skeletal muscle, and the myocardium as a basis for developing novel prevention, biomarkers, and therapies.

There is extensive evidence for signals that arise from both the adipose tissue in the form of adipokines but also from the myocardium in the form of cardiokines and cytokines (Jahng et al. 2016; Liew et al. 2017; Tang et al. 2023). Substantial literature focuses on this axis of communication from the heart to adipose tissue that exists in the form of cardiokines present in the cardiac secretome (Bermúdez et al. 2021; Tang et al. 2023). Under physiological conditions, this axis ensures homeostasis in both organs. There is also evidence that “sick” and remodeled adipose tissue, especially in the visceral fat in obesity, can influence cardiac and vascular function by the release of abnormal adipokines leading to dysfunctional cardiac remodeling, inflammation, and atrial fibrillation. These abnormal signals also induce a propensity to HF and myocardial infarction. In contrast, specific signaling from brown fat involving mitochondrial uncoupling protein has been demonstrated to be significant in offsetting cardiac stressors and ensuring metabolic stability (Challa et al. 2024). In addition, brown fat tissue has been reported to limit cardiomyocyte injury and adverse remodeling in cardiomyopathy (Thoonen et al. 2015). Extensive evidence highlights the significance of this crosstalk, detailing its role in physiology and listing numerous signaling molecules, including biochemical elements and messaging by adipokines and cardiokines. We focus here, however, on complex and poorly understood communications among the heart, adipose tissue, and β cells that depend on the activity of an isoform of the multi‐functional enzyme, p21 activated kinase (Pak1). We recognize there are other Pak1‐dependent regulatory mechanisms in vascular tissue and in the immune system (Futosi et al. 2013; Hinoki et al. 2010). However, in view of the vast literature, we decided to focus on control mechanisms in myocardium, β‐cells, and adipose tissue.

In studies summarized below over the past two decades of up and downregulation of p21‐activated kinase (Pak1) activity in cells, tissues, and animal models, we have discovered and elaborated on a previously unappreciated role of this isoform. Specifically, we have identifiedPak1 as a key regulator not only in multiple paths of control of cardiac mechanics, electrophysiology, and growth/remodeling (Ke et al. 2008, 2014; Pereira et al. 2023; Sheehan et al. 2007; Wang, Wang, et al. 2018) but also in homeostasis of adipose tissue (Batra et al. 2021; Munoz et al. 2024). Complementing these investigations is a wealth of evidence for the role of Pak1 in homeostasis of pancreatic β‐cells (Ahn et al. 2024; Huang et al. 2019; Kerr et al. 2024; Santoro et al. 2021; Umbayev et al. 2023). Here we discuss mechanisms related to the following hypothesis: Pak1 abundance and activity are significant mechanisms and points of vulnerability in the interplay among the microenvironments of the myocardium, adipose tissue, and pancreatic β‐cells.

### 1.1 Mechanisms of Activation and Effectors of Pak1 Activity

**FIGURE 1:**
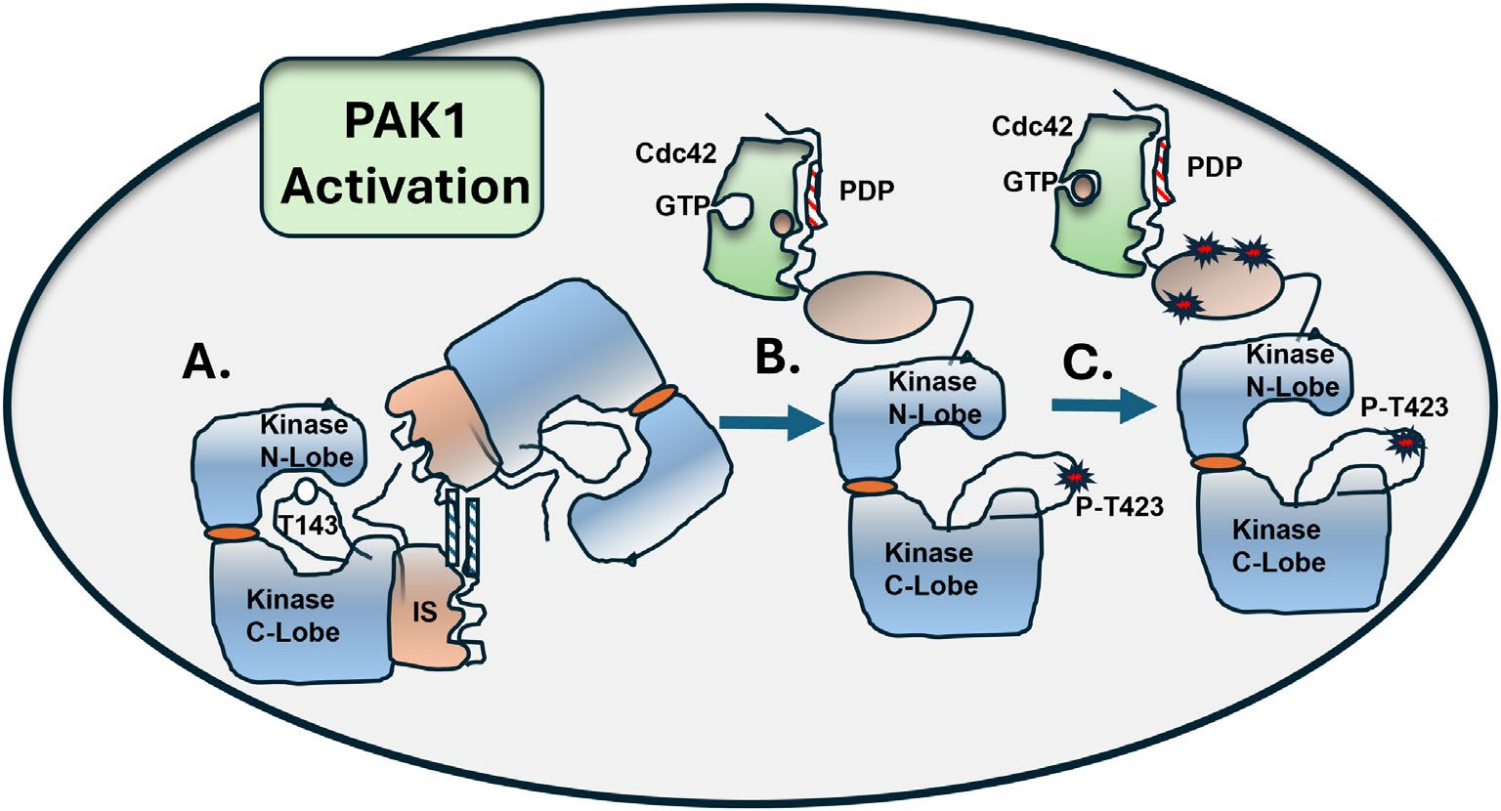
Activation of p21‐activated kinase (Pak1). In the transition to an active kinase, there is a stepwise conformational rearrangement from an autoinhibited dimeric state. (A) Inhibition is stabilized by the interaction of an inhibitory switch peptide (KI) binding tightly to a cleft in the kinase domain. (B) The dimeric configuration is disrupted by the binding of GTP‐Cdc42 or GTP‐Rac to a protein‐binding domain (PDB). This results in unfolding into an oval‐like structure with the withdrawal of the kinase inhibitory segment (KI). (C) With the phosphorylation of Thr423, there is activation of the enzyme followed by autophosphorylation of Ser sites in a stretch of 250 amino acids in the C‐terminal domain. Shown in the figure are major substrates demonstrated to be controlled by Pak1.

Paks are Ser/Thr kinases with multiple roles in regulation (Jaffer and Chernoff 2002). There are six isoforms categorized into two groups. We focus here on Pak1, which is in Group I together with Pak2 and Pak3. Studies on the role of Pak1 began in the heart with a report by Clerk and Sugden (1997) reporting high levels of Pak1 in neonatal cardiac myocytes that demonstrated an increase in enzymatic activity of Pak with hyperosmotic stress. Pak1 is abundantly expressed in the heart, adipose tissue, pancreatic β cells, and the brain (Amirthalingam et al. 2021; Ke et al. 2004; Munoz et al. 2024; Nussinov et al. 2023). Groups I and II Paks have highly conserved kinase domains at their C‐termini. N‐terminal p‐21 binding regulatory domains unique to Pak1, Pak2, and Pak3 control enzyme activity by interactions with GTP‐binding proteins Cdc42 (cell division control protein 42) or Rac1 (Ras‐related C3 botulinum toxin substrate) (Lei et al. 2000). Overlapping the p‐21‐binding domain is an autoinhibitory domain or inhibitory switch peptide (KI) uniquely expressed in Pak1, Pak2, and Pak3. As illustrated in Figure 1, in its inactive configuration, Pak1 exists as a trans homodimer with the location of the kinase domain of one monomer facing the KI of the other monomer (Figure 1). With the binding of GTP‐Rac1/GTP‐Cdc42, there is a stepwise conformational transition in which there is dissociation of the KI domain, releasing the kinase from inhibition and promoting autophosphorylation and activation of Pak1 kinase activity (Lei et al. 2005). A key phosphorylation site in the activation is Thr423, which, when pseudo‐phosphorylated, serves as a constitutively active PAK1 for adenoviral‐mediated transfer into cells (Ke et al. 2004; Manser et al. 1997).

**FIGURE 2:**
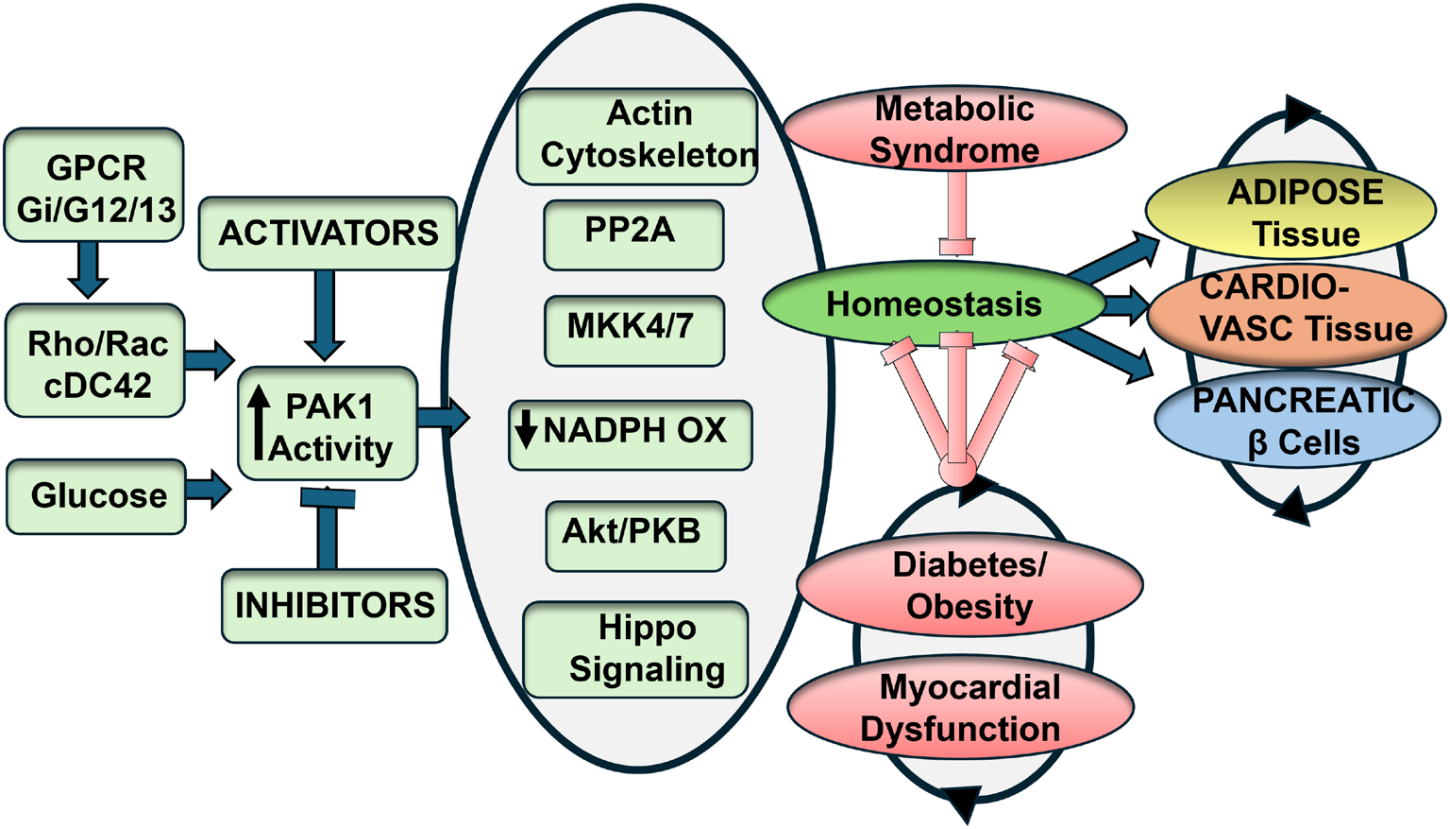
Major effectors of signaling upstream and downstream to activation of p21‐activated kinase (Pak1). This scheme of Pak1 signaling includes paths of homeostatic signaling and vulnerabilities in metabolic syndrome, obesity, Type 2 diabetes, and myocardial dysfunction. See text for further discussion.

Major substrates acting as effectors of Pak1 activation, which have been reviewed previously (Wang, Wang, et al. 2018) are also indicated in Figure 2. Signaling molecules downstream of Pak1 include the actin cytoskeleton, PP2A (protein phosphatase 2A), MKK4/7 (mitogen kinase kinase), and downstream signals via JNK (c‐Jun terminal kinase) and SRF (serum response factor) and Akt/PKB (protein kinase B). Also indicated in Figure 2 is the overlap in these pathways of signaling in the myocardium, adipose tissue, and β‐cell microenvironments. There is a path of configuration inducing scaffolding of regulators such as PP2A and Akt. Evidence for such a mechanism has been discussed in detail by Ke et al. (2013) and Kelly et al. (2013). Akt signaling represents a signaling pathway controlled by P1 with overlapping signaling in adipose tissue and the myocardium (Abeyrathna and Su 2015; Egom et al. 2011; Huang et al. 2018; Wang, Wang, et al. 2018).

### 1.2 Rationale for a Consideration of Pak1 Signaling in Signaling Among the Myocardium, Adipose Tissue, and Pancreatic β‐Cells

Our rationale in presenting this review is to inform those who have not considered Pak1 signaling in thinking about obesity/diabetes/heart disease and to inspire more research into this area of investigation. As discussed below, our lab and others have reported evidence that these disorders, which are widely recognized as linked, may involve significantly shared mechanisms of crosstalk and interplay in the form of Pak1 signaling. Depression of Pak1 levels below homeostatic levels either by whole‐body knockout or by cardiac specific deletion induces multiple cardiac abnormalities including arrhythmogenesis, altered Ca^2+^ fluxes and excitation–contraction coupling, hypertrophic remodeling, and exacerbation of ischemia/reperfusion injury. In contrast, activation of Pak1 induces stabilization of cardiac electrophysiology and protection against hypertrophic stressors such as pressure overload and elevated Angiotensin II levels (Taglieri et al. 2011). As also discussed below, more recently we discovered that reductions of Pak1 expression in global KO mice (Pak1^−/−^) induced age‐dependent increased body weight, food intake, and decreased energy expenditure with increased fat mass and decreased lean body mass (Batra et al. 2021; Munoz et al. 2024). Markers of lipogenesis increased in the Pak1^−/−^ mice. Importantly, concomitant studies of cardiac function at the whole organ and cellular level demonstrated systolic and diastolic abnormalities and evidence of arrhythmias. A corollary is that imbalances in these mechanisms result in pathological signaling (Batra et al. 2021; Munoz et al. 2024). Our hypothesis has stimulated a comprehensive consideration of the role of Pak1 in the physiology and pathology of myocardial and adipose tissue and pancreatic β‐cells with emphasis on overlapping paths of signaling among these organs. Another rationale is the recognition of the need for discussion of the effects of Pak1 in relation to the quest for the development of therapies that both stimulate and inhibit Pak1 activity. These proposed and developing targeted therapeutic strategies, particularly in cardiac dysfunction, cancer, and diabetes/obesity, require consideration and a deep understanding of Pak1 in relation to off‐target effects. Moreover, data strongly support the potential for targeted activation of Pak1 as a therapy in adverse cardiac growth and mechanics, as well as arrhythmias and for out‐of‐control adipogenesis and insulin secretion. As with the development of cancer‐related therapies, blocking the effects of Pak1, activation of Pak1 requires an emphasis on tissue‐specific targeting. For example, a confounding finding reported by Raut et al. (2015) was an upregulation of Pak1 and Cdc42 correlating with the progression of diabetic cardiomyopathy induced in rats stressed by streptozotocin treatments and 12 weeks of a high‐fat diet (HFD). However, to our knowledge, there have been no confirmations of this study.

## 2 Pak1 Signaling, Cardiac Mechanics, Cellular Ca^2+^ Fluxes, Energetics, Electrophysiology, and Remodeling

### 2.1 Pak1 as a Regulator of Excitation–Contraction Coupling in Ventricular Myocytes

**FIGURE 3:**
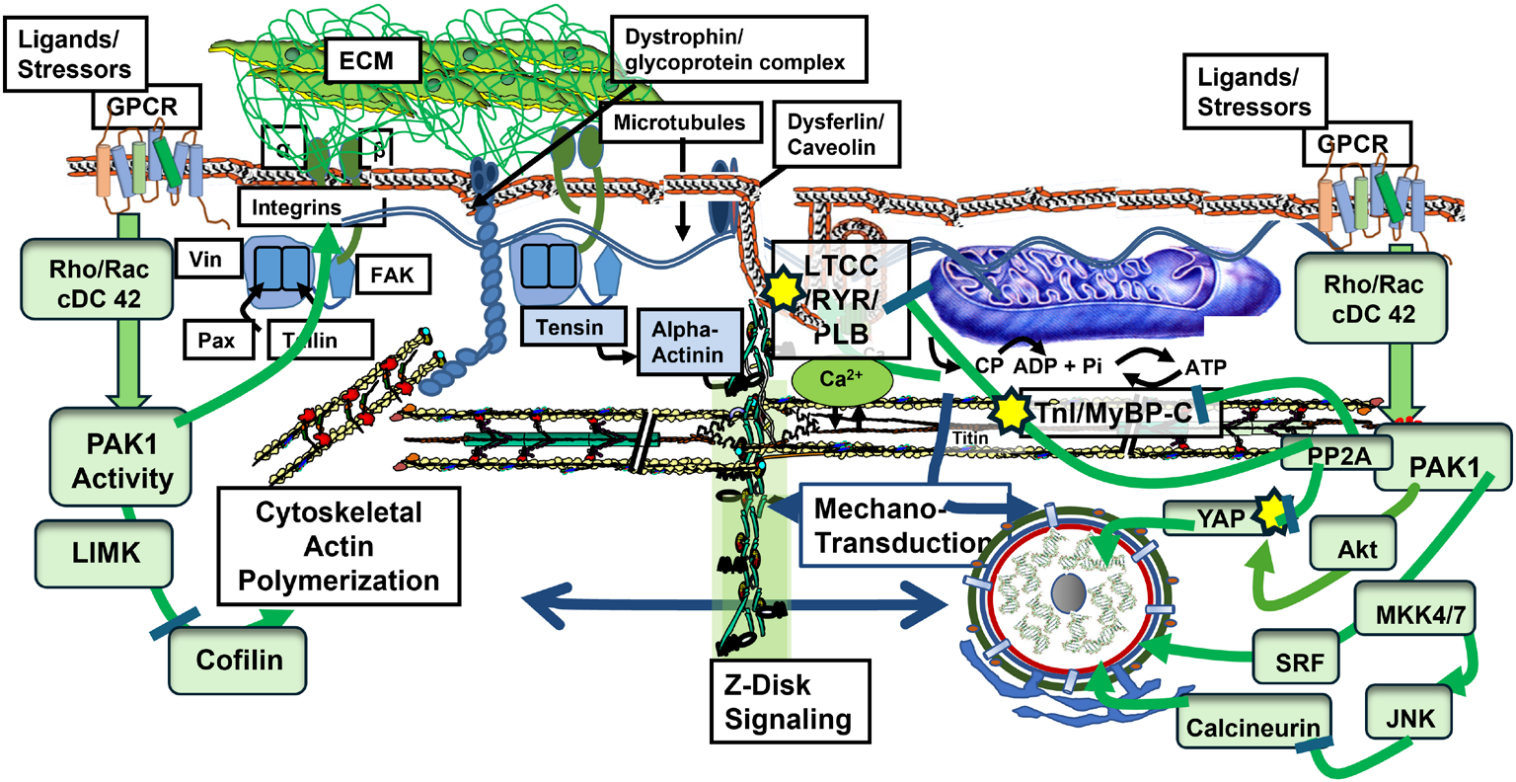
An example of the complexity of p21‐activated kinase (Pak1) signaling in a ventricular cardiac myocyte. Illustrated are the multiple pathways of signaling with the activation of Pak1. Included are signaling to actin polymerization via cofilin and LIMK (LIM kinase) and to integrins in the costamere complex, including vinculin (Vin), paxillin (Pax), focal adhesion kinase (FAK), contiguous with the extracellular matrix (ECM), and signaling networks (via tensin and tailin). Noted also is the dystroglycan/sarcoglycan complex, which is linked to these networks and dysferlin/caveolin at the t‐tubules. Other mechano‐sensitive elements are shown in a network of stress sensors, including microtubules and Z‐disk (with docked signaling molecules, one of which is Pak1 in the inactivated state). Major proteins controlling sarcomere mechanics are cardiac myosin‐binding protein C (c‐MyBP‐C) and troponin I (cTnI). The illustration depicts membrane (LTCC, L‐type Ca‐channels) and sarcoplasmic reticulum ryanodine release channels (RYR), the sarco‐endoplasmic Ca‐Pump (SERCA2a) and phospholamban (PLB) all of which control excitation and Ca‐fluxes to and from the myofilaments and area regulated by protein kinase A and protein phosphatase 2A. Green arrows indicate activating and inhibiting pathways to these cellular organelles. Also indicated is Pak1 nuclear signaling via Akt, MKK7, JNK, SRF, calcineurin, and in the Hippo pathway via YAP (yes associated protein). See text for discussion of these regulatory mechanisms and an expanded section on Hippo signaling.

To provide an example of its action in the heart, we have illustrated in Figure 3 Pak1 signaling in a cardiac myocyte. Our studies of the localization of Pak1 in ventricular myocytes demonstrated its localization at the nuclear and surface membranes as well as the Z‐disks (Ke et al. 2004). Migration of Pak1 to specific regions of the sarcoplasm was also demonstrated in sino‐atrial nodal cells (Ke et al. 2007). Early work reporting that Pak1 operates in a kinase/phosphatase signaling complex with PP2A provided a basis for our investigation of its effects on protein phosphorylation in cardiac myocytes (Westphal et al. 1999). Moreover, Buscemi et al. (2002) reported that increased Pak3 activity was able to phosphorylate cardiac troponin I (cTnI) and cardiac troponin T (cTnT) in vitro, inducing increased sensitivity to Ca^2+^ in myofilaments in detergent‐extracted fibers. These findings stimulated our lab to investigate the effects of up‐ and downregulation of PP2A and protein phosphorylation of Pak1 in the heart, where both are highly expressed (Ke et al. 2004). Figure 3 illustrates a scheme summarizing evidence for major effects of Pak1 activation at the level of the cardiac myocytes. In early studies of the effects of Pak1 on contraction and excitation of ventricular myocytes, we employed a constitutively active Pak1 (CA‐Pak1) generated by Glu substitution of Thr 423 (Manser et al. 1997). Myocytes were infected with adenovirus expressing either CA‐Pak1 or LacZ, serving as a control. The presence of CA‐Pak1 was able to increase myofilament Ca‐sensitivity in single skinned cardiac myocytes. This increased sensitivity was correlated with dephosphorylation of cTnI and with activation of PP2A. Follow‐up studies also examined the effect of CA‐Pak1 on transient changes in Ca^2+^ and tension development in single myocytes (Sheehan et al. 2009). Compared to control myocytes, those regulated by CA‐Pak1 showed slower rise times and relaxation of force under basal conditions and with isoproterenol stimulation. Spark frequency and half‐widths were also depressed in CA‐Pak1 myocytes compared to controls. These changes in dynamics were also associated with dephosphorylation of both cTnI and cMyBP‐C. These data indicated that the integrated effects of both reduced Ca^2+^ fluxes and increased myofilament Ca^2+^ sensitivity and slowing of cross‐bridge kinetics contributed to the effects of CA‐Pak1. As recently reviewed by He et al. (2023), following these early studies, a more detailed investigation of the effects of active Pak1 on cellular control mechanisms in ventricular cardiac myocytes demonstrated that, in addition to myofilament proteins, there is possible dephosphorylation of multiple membrane transporters, exchangers, and ion channels (Figure 3).

### 2.2 Pak1 in Stabilization of Ventricular and Atrial Electrical Activity

With the identification that Pak1^−/−^ results in instabilities in cardiac electrical activity and excitation–contraction coupling, we tested for its effects in maintaining electrophysiological homeostasis in ventricular myocytes in a cardiac‐specific conditional KO mouse (Pak1cko) (Wang, Tsui, et al. 2014). Mice were stressed by induction of hypertrophy by acute or chronic β‐adrenergic stimulation. We reported that the absence of Pak1 induced altered Ca^2+^ fluxes via the sarcoplasmic reticulum (SR) and reduced expression of the SR Ca^2+^‐ATPase (SERCA2a) by serum response factor (SRF). Exploration of this mechanism employing up and downregulation of Pak1 in neonatal cardiomyocytes supported the conclusion that Pak1 maintains homeostasis in the control of ventricular Ca^2+^. These findings indicate that by controlling transcription of SERCA2a, Pak1 activity acts to maintain ventricular Ca^2+^ homeostasis and electrophysiological stability.

In view of the well‐documented role of cAMP‐dependent protein kinase (PKA) regulation of pacemaker activity in the sino‐atrial node (SAN), we tested the hypothesis that promotion of PP2A activity by Pak1 slows pacemaker activity in the SAN of guinea pig hearts that express an abundance of Pak1 that co‐immunoprecipitated with PP2A. Studies inhibiting PP2A in the SAN demonstrated an increase in pacemaker activity (Ke et al. 2007). We employed adenovirus‐mediated transfer of CA‐Pak1 into SAN cells isolated from guinea pigs and intact SAN preparations. In isolated SAN pacemaker cells, our studies revealed a previously unappreciated role of Pak1 signaling in reducing the effect of isoproterenol to increase heart rate by actions at the level of the L‐type Ca^2+^ current (LCC) and delayed rectifier potassium current I_K_. Moreover, transferring CA‐Pak1 into isolated intact SAN preparations induced a significant reduction in the increase in firing rate occurring over a range of isoproterenol concentrations (Ke et al. 2007). Moreover, studies of Jung et al. (2023) have expanded the scope of understanding of protective effects in atrial arrhythmia. They reported that conditional loss of Pak1 (Pak1‐cko) expression induced atrial arrhythmias exacerbated by adrenergic challenge. Mechanisms included the demonstration of a promotion of adverse phosphorylation of CaMKIIδ and RyR2, as well as expression of Serca2a and NCX at the transcriptional level leading to Ca^2+^ overload. These findings have led to the proposal of targeting Pak1 activation as part of a novel drug class Y as proposed by Lei et al. (2018) (He et al. 2023).

### 2.3 Pak1 Activation in Cardio‐Protection Against Hypertrophic Stressors, Ischemia/Reperfusion Injury, and Cell Death

An important effect of conditional loss of Pak1 (Pak1‐cko) in ventricular myocytes is an enhanced response to stresses inducing hypertrophy, including pressure overload by aortic coarctation, chronic β‐adrenergic stimulation with isoproterenol, and treatment with Angiotensin II (Ang II) (Liu et al. 2011). Pak1^−/−^ mice were also hyperresponsive to stress induced by chronic β‐adrenergic stimulation in association with PP2A inactivation and ERK1/2 activation (Taglieri et al. 2011). Treatment of the mice with a specific inhibitor of ERK1/2 ameliorated the effect of loss of Pak1 on the ISO‐induced hypertrophic response. Pak1 also phosphorylates a specific site at Ser473 of Akt that was theorized to be important in the protection of cardiac myocytes from death (Mao et al. 2008). A demonstration of loss of this protective effect of Pak1 when Akt is inhibited provided direct support for this theory (Mao et al. 2008).

In summary, there is extensive evidence that via integrated regulation of protein phosphorylation, Pak1 activity has a critical role in the homeostasis of cardiac mechanical and electrophysiological activity, as well as physiological cardiac remodeling in exercise (Davis et al. 2015; Huang and Lei 2023; Ke et al. 2004; Wang, Tsui, et al. 2014). Activation of Pak1 is also protective in the face of cardiac stressors such as ischemia/reperfusion injury, post‐infarct heart failure, cell death, electrical instability, pressure overload (Egom et al. 2010, 2011; He et al. 2023; Liu et al. 2011, 2013; Pereira et al. 2023; Wang, Zhu, et al. 2023; Wang, Tsui, et al. 2014), and oxidative stress (DeSantiago et al. 2014, 2018). Discussed below is the protective effect of Pak1 activity in oxidative stress. Evidence of the broad implications of altered cardiac Pak1 signaling across various species is provided in a recently published study in which Zhikong scallops (*Chlamys farreri*) were stressed with heat (27°C for 30 days) mimicking global warming, which affects the vitality of this mollusk, a major food source in China (Wang, Yang, et al. 2024). Scallop hearts showed genetic evidence indicating that adverse effects of altered Pak signaling affect scallop growth, development, and reproduction.

## 3 Pak1 Signaling and Modulations in the State of Adipose Tissue and Pancreatic Β‐Cells

### 3.1 The Combined Role of Pak1 in Cardio‐Protection, Adiposity, and Energy Balance

Over the course of our investigation of Pak1^−/−^ mice, we discovered that some of the older females had weight gains suggesting increased adiposity. We therefore set out to perform a series of experiments focused on aging female mice (Batra et al. 2021). These studies identified a potential role of Pak1 at the level of the heart and the level of adipose tissue in aging female mice (Batra et al. 2021). We found that Pak1 levels increase in the hearts of middle‐aged wild‐type (WT) female mice in response to decreased Cdc42 activity. We also found Pak1 was present in visceral adipose tissue and observed that its global deletion leads to obesity, cardiac hypertrophy, and diastolic dysfunction specifically in middle‐aged female mice. Whereas the absence of Pak1 in middle‐aged Pak1^−/−^ female mice resulted in hypertrophy and impaired heart function, middle‐aged WT females appear to use increased Pak1 levels for cardio‐protection. Clinical data suggest premenopausal women have lower HF risk, which increases post‐menopause (Kuhn and Rackley 1993; Pines et al. 1992). A decline in estrogen may trigger a compensatory increase in Pak1, offering cardio‐protection. However, under certain conditions, these compensatory mechanisms may fail, resulting in overt heart failure. Our findings also indicate that middle‐aged WT female hearts show increased phosphorylated Cdc42, leading to decreased activity. Thus, increased Pak1 abundance in these hearts may serve as a compensatory mechanism to preserve cardiac function. Notably, Pak1^−/−^ mice exhibited decreased Cdc42 phosphorylation, suggesting loss of feedback regulation from Pak1. Echocardiography revealed that middle‐aged Pak1^−/−^ female mice developed concentric hypertrophy and increased LV mass, resembling human HFpEF. In contrast, middle‐aged WT females maintained normal heart function, suggesting Pak1 abundance prevents dysfunction. Additionally, middle‐aged Pak1^−/−^ female mice had the highest visceral fat mass. Overall, our findings highlight the critical role of Pak1 in preserving heart function and regulating adiposity in female mice. These sex‐specific differences point to the need for further research on Pak1's role in human heart aging and obesity, particularly in postmenopausal women.

In further studies, we explored the role of Pak1 in adipose tissue homeostasis (Munoz et al. 2024). We found that already at a younger age, Pak1^−/−^ mice show increased body weight, fat mass, and decreased lean mass. These changes were primarily driven by fat accumulation in subcutaneous white adipose tissue (scWAT), gonadal white adipose tissue (gWAT), and brown adipose tissue (BAT). The timing of these changes suggests that Pak1 impacts fat accumulation starting from early adulthood, with greater effects observed as the mice mature. Further investigation into energy balance revealed that Pak1 deletion significantly alters feeding behavior and energy expenditure. Pak1^−/−^ mice exhibited increased food intake, more frequent feeding bouts, and a greater amount of time spent feeding (Munoz et al. 2024). Interestingly, female Pak1^−/−^ mice showed a greater increase in food intake and feeding bouts compared to males, suggesting a potential interaction between estrogen and Pak1 activity in appetite regulation. Supporting this, previous studies have shown that Pak1 influences appetite through its effects on appetite‐related neurons, such as agouti‐related peptide (AgRP) neurons (Kong et al. 2016), which are known to influence feeding behavior. Additionally, we found that Pak1^−/−^ mice exhibited decreased energy expenditure, likely due to reduced thermogenesis in adipose tissues such as scWAT and BAT (Munoz et al. 2024). The decrease in thermogenesis, as indicated by lower expression of the thermogenic protein UCP1, further contributes to the observed increase in adiposity. Supporting this, Pak1's involvement in glucagon‐like peptide‐1 (GLP‐1) secretion (Grigoryan et al. 2012), which regulates glucose homeostasis and appetite, underscores its role in maintaining energy balance.

### 3.2 Pak1, Diabetes, and Insulin Secretion

As indicated above, Type 2 diabetes (T2D), which has reached and is predicted to reach epidemic levels worldwide, is a significant co‐morbidity in the interplay of obesity and heart disease (Kaiser et al. 2018; Kawai et al. 2021). In relation to our hypothesis of a role for Pak1 in this interplay, it has been reported that compared to controls, there is an ~80% decline in levels of Pak1 protein in human islets from diabetic patients, which corresponds with impaired insulin secretion (Wang et al. 2011). In the same study, Pak1^−/−^ mice showed glucose intolerance and insulin resistance. Pak1 in β‐cells promotes ERK1/2 activation for insulin release, with its absence disrupting cytoskeletal changes needed for vesicle mobilization, underscoring its role in glucose‐stimulated insulin secretion. Importantly, there is evidence that Pak1 levels and activity are involved in both β cell function and skeletal muscle glucose uptake; thus, it orchestrates multiple aspects of glucose homeostasis. Studies focused on skeletal muscle reported that Pak1 influences GLUT4 translocation via cofilin signaling. In skeletal muscle‐specific Pak1 knockout (skmPak1‐iKO) mice (Merz et al. 2022), GLUT4 translocation to the membrane was reduced, leading to lower glucose uptake and impaired insulin sensitivity. Conversely, mice with inducible overexpression of Pak1 in skeletal muscle (skmPak1‐iOE) demonstrated enhanced insulin sensitivity and glucose tolerance, with elevated PAK1 levels countering insulin resistance by preserving GLUT4 translocation (Merz et al. 2022). Beyond its role in muscle, Pak1 also facilitated “crosstalk” with pancreatic β‐cells (Merz et al. 2022). This was demonstrated using conditioned media from Pak1‐overexpressing muscle cells that boosted glucose‐stimulated insulin secretion (GSIS) in β‐cells, suggesting that Pak1 may promote the release of a signaling factor, possibly a myokine (Ryan et al. 2019), that supports β‐cell function. In human T2D skeletal muscle samples, Pak1 protein levels were reduced post‐transcriptionally, along with its effector ARPC1B (Actin‐Related Protein 2/3 Complex Subunit 1B) (Merz et al. 2022), a protein that is part of the ARP2/3 complex responsible for regulating the structure and dynamics of the actin cytoskeleton and crucial for GLUT4 translocation, suggesting that stabilizing Pak1 levels in skeletal muscle could improve glucose uptake and help manage insulin resistance in T2D patients.

To investigate the role of Pak1 in pancreatic β‐cells, a β‐cell specific inducible Pak1 KO (βPAK1‐iKO) mouse model was generated (Ahn et al. 2021). Key findings indicate that Pak1 is essential for maintaining the mitochondrial electron transport chain (ETC) integrity, especially in preserving protein levels of Complexes I, III, and IV. This study identified NDUFA12 (NADH: ubiquinone oxidoreductase subunit A12), a subunit of mitochondrial complex I, as a critical protein regulated by Pak1 in pancreatic β‐cells. Moreover, the loss of Pak1 in β‐cells led to impaired glucose‐stimulated insulin secretion, reduced mitochondrial respiration, and increased ROS, ultimately resulting in β‐cell apoptosis. This study also showed that replenishing Pak1 in diabetic human islets alleviates ER stress and improves β‐cell function. These results suggest that Pak1 protects β‐cells from stress‐induced apoptosis by stabilizing mitochondrial ETC proteins and regulating redox balance.

Building on this understanding of Pak1's protective effects, its role under diet‐induced stress conditions was further explored using a doxycycline‐inducible mouse model that overexpresses Pak1 specifically in β‐cells (iβPak1‐Tg) (Ahn et al. 2024). iβPak1‐Tg fed a high‐fat diet (HFD) showed improved glucose tolerance, increased insulin content, and reduced β‐cell apoptosis compared to controls. The study also showed that Pak1 plays a role in insulin biogenesis. Pak1‐enriched β‐cells had increased expression of transcription factors like PDX1 (pancreatic and duodenal homeobox 1) and NEUROD1 (neuronal differentiation 1), both of which are essential for insulin gene transcription. Pak1 appears to facilitate these factors' binding to the insulin gene promoter, enhancing insulin synthesis and β‐cell function. This suggests that Pak1 not only protects β‐cells from stress but also promotes the cellular machinery required for sustained insulin production. Taken together, these studies highlight the critical role of Pak1 in regulating glucose metabolism, insulin sensitivity, and β‐cell survival, positioning Pak1 as a promising therapeutic target for T2D in addition to its promise as a target for obesity and cardiac dysfunction.

## 4 Pak1 Signaling in Protection Against Oxidative Stress in the Myocardium, Adipose Tissue, and β‐Cells

### 4.1 Pak1 Activity and Protection of Cardiac Function in Oxidative Stress

Studies by Banach and colleagues (2014, 2018, 2023) on the role of Pak1 activity as a factor in SAN dysfunction and atrial fibrillation (AF) focused on the effect of loss of activity of Pak1 to result in an increase in the activity of NADPH oxidase 2 (NOX2) to generate reactive oxygen species (ROS). These studies were the first to identify the inhibition of NOX2 by Pak1 in the myocardium. Experiments supported the hypothesis that the ROS generation was indeed due to increased activity of NOX2 with the loss of Pak1. These studies showed the effects of loss of Pak1 to reduce the expression of the hyperpolarization‐activated cyclic nucleotide‐gated cation channel (HCN). Interestingly, the ROS scavenger TEMPOL and Class II HDAC inhibition eliminated the effects of the loss of Pak1 and restored the HCN activity to the same level as in wild‐type hearts.

### 4.2 Pak1 Activity and Protection of Pancreatic β‐Cells and Adipose Tissue in Oxidative Stress

Several NOX isoforms are expressed in β‐cells (Kowluru 2010). In diabetes, the induction of ROS occurs with an imbalance of free radical scavenging and metabolic activity as well as the expression of pro‐inflammatory cytokines, elevated free fatty acids, and glucose (Weaver and Taylor‐Fishwick 2013). The prominent role of Pak1 signaling in β‐cell function indicates that this oxidative stress would affect actin signaling and the Hippo pathway in the pancreas. An important question is whether activation of Pak1, as is the case in the myocardium, would protect β‐cells from the adverse effects of ROS. Evidence for Pak1 activity, which is also a significant factor in mitochondria‐generated ROS in β‐cells, was obtained in experiments with mice harboring a pancreatic β‐cell‐specific inducible Pak1 KO (βPak1‐iKO) (Ahn et al. 2021). Compared to controls, βPak1‐iKO mice showed intolerance to glucose, which, at the cellular level, showed loss of insulin secretion and mitochondrial dysfunction with loss of abundance of mitochondria and disruption of the NAD^+^/NADH and NADP^+^/NADPH ratios leading to elevated ROS. Restoration of Pak1 levels in the βPak1‐iKO restored normal stress markers. Thus, a main conclusion of these studies was that Pak1 has a protective role in maintaining mitochondrial homeostasis in human β‐cells (Ahn et al. 2021). It is relevant that ROS‐related pathogenesis occurs in prediabetes with altered mitophagy and mitochondrial dynamics (Veluthakal et al. 2024).

Oxidative stress associated with adiposity is a well‐documented important contributing factor to the pathological progression of metabolic syndrome (Rani et al. 2016; Świątkiewicz et al. 2023). Importantly, in our consideration of crosstalk between the myocardium and adipose tissue, there is evidence that when controlled, physiologically, ROS modulates healthy WAT expansion and function. When ROS is out of control, expansion of WAT occurs with metabolic dysfunction (Jornayvaz et al. 2024). Major sources of oxidative stress in adipose tissue are mitochondria and NOX‐related generation of ROS. With the increased adiposity in obesity, there is a significant increase in ROS generation, as confirmed by biomarker studies in humans, animal studies, and in vitro determinations in isolated fat cells (Le Lay et al. 2014). The increase in WAT in obesity contributes to an increase in mitochondrial load, with a resultant increase in nutrients as substrates for oxidative phosphorylation, with the generation of oxygen free radicals and ROS (Le Lay et al. 2014). Evidence indicates that oxidative stress induces dysfunction in the vascular/endothelial compartment, as well as systemic inflammation (Świątkiewicz et al. 2023). As summarized above, Pak^−/−^ mice demonstrate an increase in WAT, indicating the potential for mitochondrial dysfunction and ROS (Batra et al. 2021; Munoz et al. 2024). Demonstrations that Pak1 kinase activity is protective against oxidative stress provide evidence in support of the potential therapeutic impact of Pak1 activation. Induction of dysfunction of pancreatic β‐cells also involves adverse effects of oxidative stress (Kowluru 2010).

Studies of mice stressed by induction of obesity in a HFD have confirmed a connection between adiposity and AF and provided new insights into ROS generation and regulatory pathways. Recognizing the connection between ROS and AF, Sridhar et al. (2024) investigated a role for the activation of NOX2 in this relationship. They compared controls and *Nox‐2‐knockout* mice stressed by obesity‐induced shortening of the atrial action potentials, a hallmark of AF. They also investigated action potential remodeling in human atrial myocytes generated from induced pluripotent stem cells (hiPSC‐atrial CM). The action potential remodeling occurred with palmitic acid and H_2_O_2_ treatment with and without inhibition of NOX2. This line of experiments was also carried out in human primary atrial preparations. These experiments confirmed the connection between obesity and action potential remodeling as well as structural remodeling indicated by reduced mRNA expression of connexin 40 by *GJA5*. A novel finding from the investigation of transcriptional profiles was the demonstration that indicated the linkage of an upregulation of the pleiotropic gene PITX2 (paired‐like homeodomain transcription factor 2) to action potential remodeling and this structural remodeling and NOX2 upregulation. Earlier studies reported that a loss of PITX2 in hIPSC‐atrial myocytes mimics the action potential remodeling in AF (Schulz et al. 2023). These studies indicated that levels of PITX2 expression are responsible for the electrical abnormalities in AF but did not make a connection with obesity and NOX2. It is also significant that other recent studies have identified SNPs (single nucleotide polymorphisms) in PITX2 related to AF and SAN dysfunction (Yan et al. 2025).

Despite the extensive evidence for a protective role of Pak1 activation in ameliorating stress‐induced ROS in the context of experiments described above, an opposite conclusion has been discussed in an extensive review by Guo et al. (2022). Their evaluation of the literature included evidence that loss of Pak1 induced oxidative stress, but they concluded that Pak1 signaling promotes ROS generation, suggesting that inhibition of Pak1, not activation, would be therapeutically effective in cardiotoxicity. This review indicating that inhibition of Pak1 may be effective in cardiovascular disorders is also reflected in earlier reviews and provides an indication of the complexity of modulation of Pak1 and the need for further understanding of the complexity of its signaling pathways and the development of specific therapeutic modifications that are appropriate for the context of the disorders in the cardiovascular system (Amirthalingam et al. 2021; Kelly et al. 2013).

## 5 Pak1 and the Actin Cytoskeleton

### 5.1 Pak1 and Regulation of the Myocardium, Adipose Tissue, and β‐Cells

Promotion of the Cdc41 and Rac1 signaling cascade, which is a well‐known regulator of the actin cytoskeleton, activates Pak1, thereby influencing several downstream pathways that control actin polymerization and reorganization (Sussman et al. 2000; Brown et al. 2006; Edwards et al. 1999). There is evidence that actin phosphorylation and cytoskeletal reorganization occur with the association of PI‐3 kinase with Pak1 (Papakonstanti and Stournaras 2002). In this signaling path, Pak1 is activated and phosphorylates actin independently of GTP‐Rac1/GTP‐Cdc42. Non‐sarcomeric actin filaments (F‐actin) are dynamic structures that undergo constant assembly (polymerization) and disassembly (depolymerization), which is critical for cellular processes like migration, shape maintenance, intracellular trafficking, and mechano‐transduction (Henderson et al. 2017). In cardiac myocytes, this mechano‐transduction is critical for homeostatic stability and as a vulnerable process in disorders such as cardiomyopathies and pressure overload (Figure 2) (Grimes et al. 2019; Hoshijima 2006). The state of the actin cytoskeleton is also important in the movement of vesicles such as insulin granules or glucose transporter 4 (GLUT4) vesicles (Tong et al. 2001). Related to the role of Pak1 in metabolic control are data indicating that Cdc42 is activated by glucose in pancreatic islet cells, resulting in the activation of Pak1 upstream of the activation of Raf‐1, MEK1/2, and ERK1/2. This signaling path leads to F‐actin remodeling and the induction of insulin release by recruiting insulin granules to the surface membrane (Kalwat et al. 2013). The remodeling of F‐actin in response to insulin, rather than just its polymerization, is crucial for the movement of GLUT4 storage vesicles (GSVs) to the plasma membrane in skeletal muscle cells (Tong et al. 2001; Zaid et al. 2008). Furthermore, Pak1 plays a role in both the polymerization and depolymerization of F‐actin during insulin‐stimulated GLUT4 vesicle translocation and glucose uptake in L6‐GLUT4myc myoblasts and myotubes (Tunduguru et al. 2014, 2017). In studies reviewed by Jeon et al. (2023), enlarged lipid droplets in adipocytes were associated with downregulated F‐actin formation and impairment in insulin‐dependent GLUT4 trafficking. Enlarged lipid droplets can lead to reduced fat breakdown (lipolysis). This means that the body has difficulty mobilizing stored fat for energy, contributing to further fat accumulation. In our Pak1^−/−^ studies, we found enlarged adipocyte size and decreased lipolysis, and increased lipogenesis gene expression in WAT (Munoz et al. 2024). Given its role as a key regulator of actin polymerization and cytoskeletal dynamics, Pak1 activity may play a role in lipid droplet formation. Lipid droplets, as dynamic organelles, undergo constant remodeling, including processes like fusion and fission, which are influenced by cytoskeletal components such as F‐actin. By promoting F‐actin polymerization, Pak1 may influence lipid droplet dynamics, affecting their formation, size, mobility, and distribution within the cell. In cells with active Pak1 signaling, smaller and more mobile lipid droplets may be generated due to efficient actin‐dependent trafficking. Conversely, when Pak1 signaling is disrupted, the trafficking of lipid droplets may be impaired, leading to the accumulation of enlarged lipid droplets. Enlarged lipid droplets are often associated with insulin resistance, and as Pak1 is crucial for maintaining insulin sensitivity and glucose metabolism, its dysfunction could also contribute to lipid droplet enlargement and adipocyte hypertrophy. Altogether, these mechanisms may contribute to overall metabolic health, potentially exacerbating metabolic disorders when Pak1 signaling is impaired. In the following sections, we discuss a role of Pak1 in cytoskeletal dynamics associated with control of the Hippo pathway.

### 5.2 Interplay and Crosstalk Between Oxidative Stress and the Actin Cytoskeleton

A relation between RhoGTPase Rac1 in actin cytoskeletal dynamics and the generation of oxidative stress constitutes a convergence of signals present in the myocardium, adipose tissue, and β‐cells (Acevedo and González‐Billault 2018). This convergence occurs because both NOX and cytoskeletal dynamics are controlled by the same upstream small G protein. ROS generated via NOX may directly alter actin cysteine phosphorylation, altering cytoskeletal function and its role in Hippo signaling. Evidence from experiments with gain and loss of Pak1 activity summarized above emphasizes the potential significance of Pak1 signaling in myocardial dysfunction, adiposity, and β‐cell function. Functions in all three of these tissues are altered by oxidative stress that implicates association with Pak1 signaling and the Hippo pathway. In Section 4.1, we presented accounts of evidence of a role for Pak1 in the stabilization of myocardial function with a role as a protector against oxidative stress. Below we discuss the role of the cytoskeleton in Hippo signaling and evidence that NOX activity may influence Hippo signaling by affecting actin dynamics. Additionally, we examine how the activation of Pak1 may offset this adverse signaling.

## 6 The Interface of Pak1 Signaling and the Hippo Pathway in Reciprocal Communications Among the Myocardium, Adipose Tissue, and β‐Cells

So far, we have presented our data and related evidence in the literature supporting the hypothesis that elements of Pak1 signaling are common in homeostasis and pathologies in the myocardium, adipose tissue, and β‐cells and thus important to understand in the context of obesity, diabetes, and heart diseases. We next discuss evidence of convergence of these pathways with Hippo signaling. The Hippo pathway has been conserved throughout evolution as an overarching signaling cascade in the hierarchy of tissue regulation (Ardestani et al. 2018; Langa et al. 2022; Wu et al. 2024; Yu et al. 2015). Hippo signaling interrogates the microenvironment of many tissues by surveillance of an array of intracellular and extracellular signals, alterations in cell contacts and polarity, mechanical and contractile state, G‐protein coupled receptor–ligand binding, and the state of cellular energy (Jafarinia et al. 2024; Kim and Gumbiner 2015; Piccolo et al. 2014; Zhao, Li, et al. 2012). Although these cited reviews and publications include elements of crosstalk of Hippo signaling with other paths of signaling, most do not explicitly include Pak1, despite abundant evidence of its potential involvement. We focus here on prominent regulatory elements potentially overlapping with the Hippo cascade and Pak1 signaling. These include PI3/Ca/Akt signaling, signaling via NAPH oxidases, cytoskeletal signaling, neurofibromin 2 (NF2, also known as Merlin), and sphingolipid signaling. There are excellent reviews addressing the role of Hippo signaling in cells in the cardiac microenvironment and broad reviews of Hippo signaling in health and disease (Fu et al. 2022; Hu et al. 2024; Yu et al. 2015). However, these reviews do not focus on mechanisms of Hippo modulation in inter‐organ communication and do not discuss the role for Pak1 in this communication. Moreover, consideration of Hippo modulation in insulin secretion in β‐cell function and in the homeostasis of adipose tissue has not been included in a recent extensive review on Hippo signaling in cell types in the myocardium (Hu et al. 2024). Studies in lung tissue demonstrate a role for Hippo signaling in intercellular communications (Govorova et al. 2024). The crosstalk between Hippo signaling involved in the homeostasis of lung cell signaling provides an excellent example of the potential for similar paths of signaling in the homeostasis of adipose and heart tissue and communication between these tissues in homeostasis and dysfunction. The authors summarized crosstalk in the lung involves Hippo signaling with WNT, SHH, TGFβ, Notch, Rho, and mTOR, but with no mention of Pak1, which is undoubtedly involved in the paths described in the paper. Thus, one rationale for our discussion is a general lack of inclusion of Pak1 in considerations of Hippo signaling. There is also a lack of consideration of Hippo/Pak1 signaling in inter‐organ communications as described here.

### 6.1 Hippo Signaling and Promotion of Homeostatic Nuclear Signaling

**FIGURE 4:**
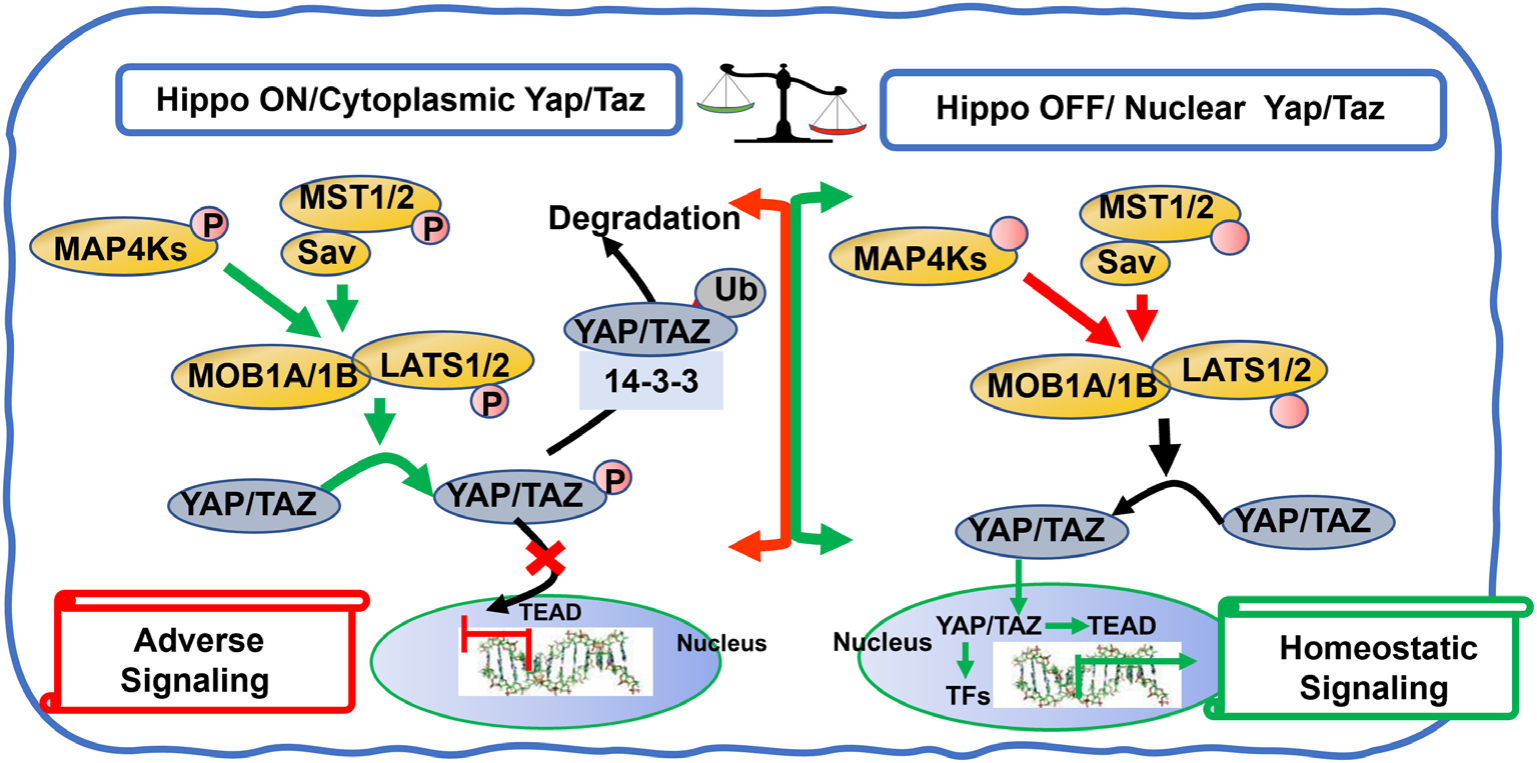
Scheme illustrating ON and OFF states of the canonical Hippo signaling pathway. The right panel of the scheme depicts evidence summarized in the text. Demonstrating in the basal homeostatic OFF state, there is relative suppression or restraint of phosphorylations in the Hippo pathway, which permits the nuclear transcriptional activator complex Yap (yes associated protein) and Taz (transcriptional coactivator with PDZ‐binding motif) to move from a cytoplasmic location into the nucleus, relieving transcriptional repression by TEAD (targeting transcriptional enhanced associate domains). As more fully discussed in the text and summarized in Figure 5, this transcriptional activation is important in the homeostasis of the physiologically stable state of the myocardium, adipose tissue, and pancreatic β‐cells, as indicated by the scroll at the right side of the scheme. Evidence indicates that in dysfunction in these tissues, there is a shift in the balance to the Hippo “ON” or unrestrained state, shown in the left panel, in which phosphorylated Yap is retained in the cytoplasm in a process that depends on a cascade of upstream phosphorylation signals. Activation of Mst1/2 (sterile 20‐like kinase 1 and 2) in combination with Sav (scaffolding protein Salvador) results in the activation of Lats1/2 (large tumor suppressor 1 and 2) that, in combination with MOB1A and 1B (Mps one binder 1A and B) promote phosphorylation of Yap/Taz, preventing its entry into the nucleus. In a dominant ON state, there is suppression of nuclear signaling promoting dysfunction, as listed as indicated in Figure 5. As indicated in Figure 1, the cytoplasmic Yap may sequestrate in the cytoplasm or be removed by ubiquitin‐related mechanisms involving specific forms of 14‐3‐3 proteins.

**FIGURE 5:**
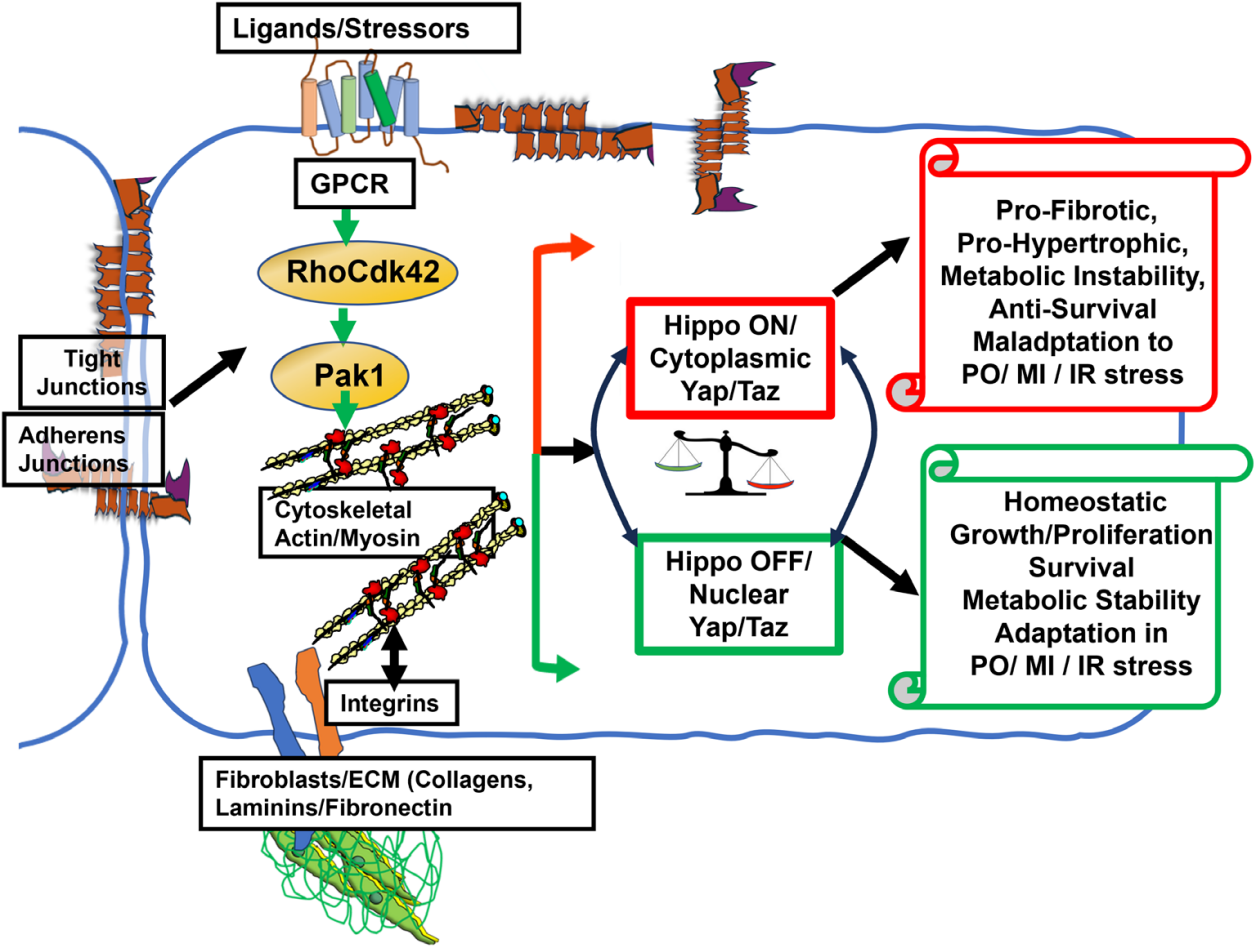
Scheme illustrating signals modulating ON and OFF states of the canonical Hippo signaling pathway. As shown and discussed in the text, signals engaging the Hippo pathway include GPCR activation by ligands, mechano‐signaling via the actin cytoskeleton/stress fibers, and the state of tight and adherens junctions. Other elements of Hippo mechano‐signaling include integrins, the ECM including collagen isoforms, and glycoproteins (fibronectin and laminins).

Figure 4 illustrates essential components of core canonical Hippo signaling, which are present in multiple tissues including the micro‐environments of cardiac myocytes, adipose tissue, and pancreatic β‐cells (Ardestani et al. 2018; Ardestani and Maedler 2018; Hu et al. 2024). As shown in Figure 4 there are two states of activity of the Hippo pathway described as OFF (in reference to upstream activators) with unrestrained nuclear translocation of the co‐activators YAP (Yes associated protein) and Taz (transcriptional coactivator with PDZ‐binding motif) that leads to promotion of TEAD transcriptional activation. Extensive evidence over the years indicates that in the basal homeostatic state there is suppression of phosphorylation of the nuclear transcriptional activator complex. In their unphosphorylated state YAP and TAZ move from the cytoplasmic to the nuclear compartment relieving transcriptional repression by TEAD (targeting transcriptional enhanced associate domains). The promotion of transcription by YAP/TAZ induces expression of multiple effectors of cell proliferation, survival, hypertrophic growth, anti‐fibrotic signaling, anti‐apoptotic signaling, and mitochondrial functional stability (Hu et al. 2024). In a state of ON (in reference to upstream signaling) there is a phosphorylation of YAP/TAZ leading to their cytoplasmic retention and restrained nuclear translocation leading to suppression of TEAD transcriptional activation. These phosphorylations are promoted by activation of MST1/2 (sterile 20‐like kinase 1 and 2) in combination with SAVF (scaffolding protein Salvador) that results in activation of LATS1/2 (large tumor suppressor 1 and 2), which in combination with MOB1A and 1B (Mps one binder 1A and B) promote phosphorylation of YAP/TAZ. Levels of YAP/TAZ in the cytoplasm are also modulated by ubiquitin‐related removal mechanisms involving specific forms of 14‐3‐3 proteins. Robust Hippo/YES/TAZ induced transcriptional activation is important in organ development. In the adult low levels of transcriptional activation are important in homeostasis of mature organs. As illustrated in Figure 5 this transcriptional activation is important in homeostasis of cardiac function and structure, vascular and endothelial stability, and homeostatic stability of the extracellular matrix. For example, cardiac specific knock‐out of TEAD in cardiac myocytes in mice resulted in adverse effects on regulation by the sarcoplasmic reticulum, sarcomeres and mitochondrial dysfunction (Liu et al. 2017). This same study reported low levels of TEAD expression in human failing hearts.

**FIGURE 6:**
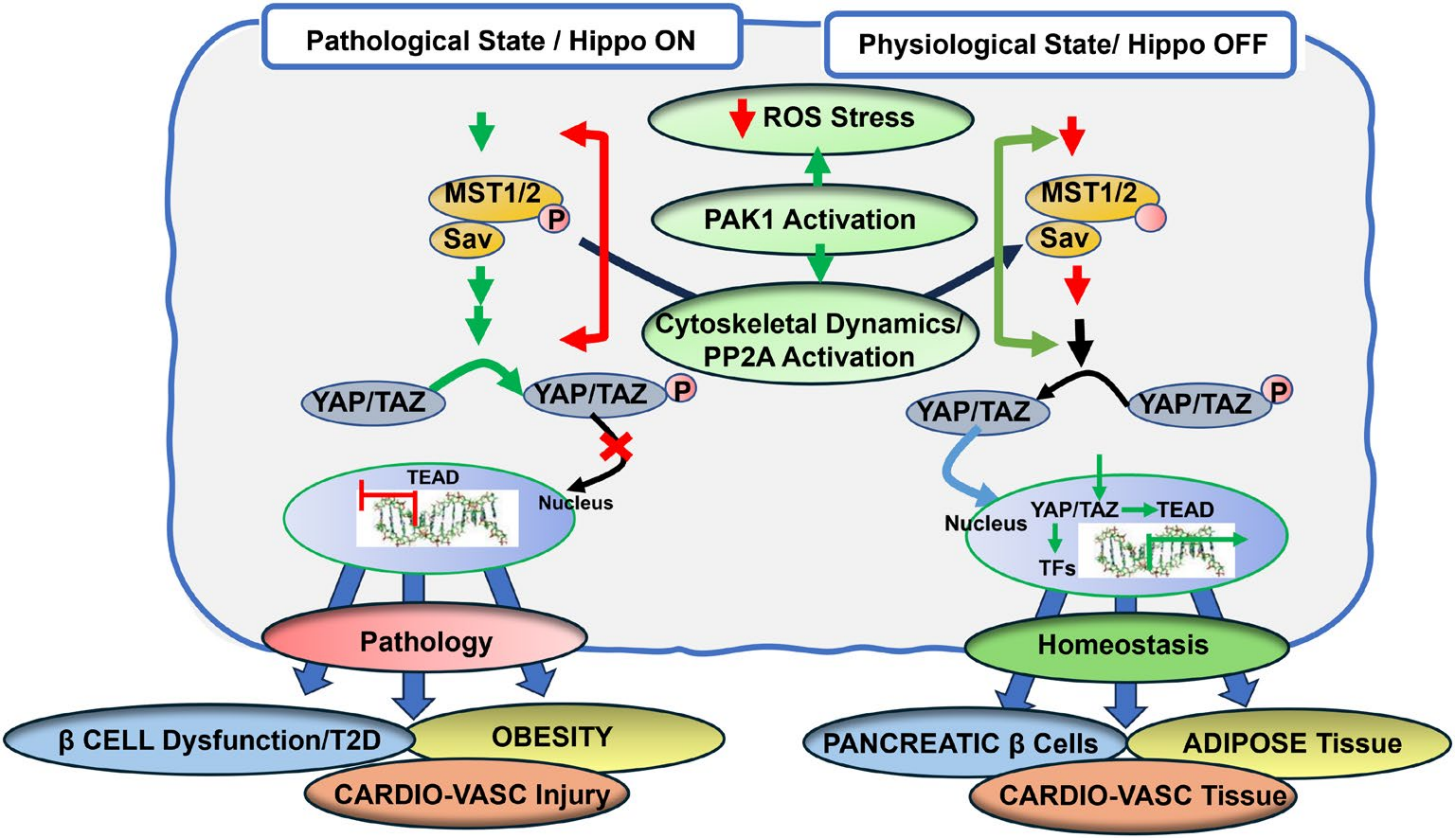
Scheme illustrating potential mechanisms by which activation of the p21 activated kinase (Pak1) may shift Hippo signaling to an OFF state suppressing pathological signaling by modifying upstream signals to Yap/Taz. As shown, signals engaging the Hippo pathway include GPCR activation by ligands and mechano‐signaling that shift from ON to OFF states. Shown are the effects of Pak1 activation to shift to an OFF state in which homeostasisis restored.

Mathematical models of Hippo signaling that include crosstalk with other regulatory mechanisms have provided an excellent framework for this discussion (Jafarinia et al. 2024; Khalilimeybodi et al. 2023). These models employ modules that aid in providing the bookkeeping necessary for computational approaches. The model includes the following modules: IP3‐Ca, F‐actin, RhoA, Kinase, and GPCRs. Although the models include signaling that affects Pak1 signaling or are affected by Pak1 signaling, there is no inclusion of its activity in the modeling. We have therefore concisely extended the description of these modules to include Pak1 (Figures 4, 5, 6).

### 6.2 Regulatory Mechanisms Common to Pak1 Signaling and Hippo Signaling

Significant mechanisms that control ON/OFF Hippo signals are likely to involve an interplay with Pak1 signaling. Both Pak1 and Hippo signaling share common regulatory cascades. These include Rho Kinase/Cdc42 signaling, cytoskeletal reorganization (Meng et al. 2016), and control of phosphorylation of kinases upstream to YAP/TAZ. In an analysis of mechano‐transduction mechanisms, it is important to note the complexity of the ECM, which, as indicated in Figure 5 includes signaling via collagens, laminins, and fibronectin. An example of specificity in the ECM signaling cascades is Tank‐binding protein kinase‐1 (TBK1), which has been reported to regulate pathways that control fibroblast activation via specific cytoskeletal proteins (Aravamudhan et al. 2020). Reduction of TBK1 expression in fibroblasts induced reductions in YAP/TAZ levels and was associated with reductions in α‐smooth muscle actin stress fiber levels and increasing deposition of ECM collagen 1 and fibronectin. Moreover, altered mechanical cues affecting Hippo signaling led to activation of TBK1. There is also evidence that fibronectin adhesion signaling suppresses the Hippo pathway, leading to YAP nuclear localization (Kim and Gumbiner 2015). Jang et al. (2020) reviewed the relations between the Hippo pathway and small GTPase pathways in the regulation of YAP/TAZ. Their summary included a discussion of Pak1 in the signaling cascades. This interplay of regulation indicates that modulation of signaling by various techniques such as overexpression/deletion of Pak1 or pharmacological activation may affect Hippo signaling. This is confirmed in inhibition of Pak1 in cancer pathologies in which there is an interplay between Hippo signaling and Pak1 activity. Here we discuss the potential role of Pak1 activation acting in concert with Hippo signaling and resulting in improvements in myocardium, adipose tissue, and pancreatic β‐cell function in response to mechanical and biochemical stresses. Figure 6 illustrates this therapeutic approach. We next discuss evidence supporting the confluence of Pak1 and Hippo signaling. These mechanisms common to both cascades are relevant to therapeutic strategies involving activation and inhibition of Pak1 activity.

As illustrated in Figures 4, 5, 6, a prominent signaling pathway sensed by the Hippo pathway and promoting Pak1 activation includes mechano‐signaling and GPCR signaling via Rho, Cdc42, and the actin cytoskeleton. This signaling is significant in the relay of mechanical and ligand signals sensed by tissues as an effector of YAP/TAZ nuclear localization leading to altered gene expression. Interactions between the ECM and variations in ECM stiffness, together with cytoskeletal tension, have been demonstrated to modulate YAP nuclear localization (Dupont et al. 2011; Kim and Gumbiner 2015; Zhao, Li, et al. 2012). Important insights into potential relations between Pak1 and Hippo signaling were reported in early studies demonstrating in isolated cell systems that constitutively active Pak1 induced a loss of stress fibers associated with a loss of focal adhesions (Field and Manser 2012; Manser et al. 1997). These authors concluded that PAK is important in the dissolution of stress fibers and focal complex reorganization. Although Hippo signaling is not mentioned in these papers, later studies, as reviewed by Dupont (2016) summarized the important role of the mechanical state of the ECM and focal adhesions in signaling Yap/Taz in the Hippo pathway. Adhesion in cell–cell interactions also modulates YAP nuclear localization, enhancing cell rigidity and stability in a manner dependent on the actin cytoskeletal state as well as the PI3K kinase pathway (phosphatidylinositol 4,5‐bisphosphate 3‐kinase) (Kim and Gumbiner 2015; Zhao, Li, et al. 2012). In contrast, when cells are detached from their neighbors, Hippo signaling is ON, resulting in suppression of YAP nuclear localization and detached cell death (anoikis) (Zhao, Li, et al. 2012). Hansson et al. (2019) reported that adipocyte size correlates with a significant remodeling of the actin cytoskeleton. A HFD fed to mice for 2 weeks induced an expansion of adipocytes with increased abundance of actin filaments. Associated with this induction was increased Rho‐kinase activity and altered regulation of actin. Other studies demonstrated that a HFD‐induced increased expression of Rho‐kinase with increased adipocyte size, gain in weight, and glucose intolerance (Hara et al. 2011). These changes were reversible with the inhibition of Rho‐kinase (Hara et al. 2011). Interesting mechano‐signaling stimulated by cell stretch also induced expression of Rho‐kinase. The authors speculate that the hypertrophic growth of the adipocytes induces a stress‐sensing mechanism resulting in increased Rho‐kinase expression. Recent studies demonstrated that Rho/ROCK expression varies depending on location, with differences noted in the expression of perigonadal WAT and brown adipose tissue (Muñoz et al. 2024). An important observation was that there was reduced ROCK activity with exercise in adult and middle‐aged rodents and humans.

### 6.3 Hippo Signaling, Pak1, and the Myocardium

We have previously published a review on a role for Hippo signaling in dilated cardiomyopathy (DCM) linked to mutant cardiac myofilament proteins encoded by variants of sarcomere‐expressing genes (Langa et al. 2022). We discussed Hippo signaling instigated by the altered mechanical state associated with the expression of variants of sarcomere proteins linked to DCM. Modulation of Mst1 in Hippo signaling in myocardial I/R injury and heart failure provides salient examples of potential mechanisms by which Pak1 activation may be cardio protective, as described above. Figure 6 illustrates our proposal for this action of Pak1, which indicates that the activation of Mst1 signaling cardiac stressors such as ischemia/reperfusion injury, oxidative stress, heart failure, and HCM is offset by Pak1 activation. In response to these stressors, Mst1 kinase is activated by either or both autophosphorylation and cleavage by caspase (Ma et al. 2019). To determine the significance of this increase in Mst1 kinase activity in the canonical Hippo pathway, investigations have employed knockdown/ablation approaches or expression of a dominant negative Mst1 in stressed mouse models (He et al. 2024; Hu et al. 2024; Odashima et al. 2007; Yamamoto et al. 2003). A cogent example is studies stressing hearts by ischemia or I/R or oxidative stress in with downregulation of Mst1 kinase activity, there was diminished cell death, fibrosis, and expression of cytokines (Odashima et al. 2007; Yamamoto et al. 2003). Moreover, overexpression of Mst1 in a mouse model induced dilated cardiomyopathy (DCM) associated with enhanced YAP phosphorylation, as well as mitochondrial and metabolic dysfunction (Wu et al. 2021). Another cogent example is the suppression of isoproterenol‐induced adverse functional changes by cardiac‐specific ablation of Mst1 in a mouse model (He et al. 2024). There was inhibition of apoptosis, oxidative stress, and Ca^2+^ overload, as well as mitochondrial dysfunction and attenuation of the release of cytokines in an inflammatory response. It was also noted in bioinformatic analysis of the Mst1^cko^ hearts that there was an enrichment of the FoxO (Fork head box protein) pathway. Interestingly, it has been reported that the activation of Mst1 kinase by I/R may also function in the non‐canonical Hippo pathway by site‐specific phosphorylation of FoxO1 and c/EBP‐β (CCAAT‐enhancer‐binding proteins) (Maejima et al. 2024). Importantly, it was demonstrated that this phosphorylation may occur without activation of Mst1 kinase activity, raising the possibility of promoting cell survival by Mst1 while diminishing its adverse effects.

### 6.4 Hippo Signaling, Pak1, Obesity, and Insulin

Literature supporting a role for cDC42 and therefore Pak1 signaling as a critical component of insulin secretion and diseases related to diabetes has been summarized in extensive reviews (Huang et al. 2019; Kowluru 2017). These reviews indicate that under physiological conditions, Cdc42 expression controls essential functions, including cytoskeletal remodeling, transport and fusion of vesicles, as well as insulin secretion and proliferation of β‐cells. These functions of Cdc42 are also important in glucose‐stimulated insulin secretion. Importantly, dysregulation of Cdc42 in diabetes progression participates in insulin resistance, diabetic neuropathy, and metastasis of tumors under hyperglycemic conditions (Kowluru 2017). An example of the role of Cdc42/Pak1 signaling in age‐related obesity and insulin signaling has also been reviewed (Umbayev et al. 2023). The review of these regulatory processes revealed a possible relation among age‐related obesity and metabolic dysregulation, insulin/leptin signaling, and signaling via Cdc42/Pak1. The authors propose that inhibition of paths of Cdc42 signaling may be therapeutic for obese patients.

There is also evidence that Hippo/Mst signaling promotes the terminal differentiation of adipocytes (Park et al. 2012). The effector of this signaling is PPARγ (peroxisome proliferator‐activated receptor) which is an important regulator of adipogenesis through the activation of adipocyte gene regulation (Gerhold et al. 2002). The activation of PPARγ involves a stabilization by interactions with the scaffolding protein SAV1 and the kinase activity of Mst (Gerhold et al. 2002). In the case of adipocyte proliferation and differentiation, it has been reported that LATS2‐dependent phosphorylation of YAP/TAZ retains these factors in the cytoplasm, permitting PPARγ transcriptional activity. Together with other regulatory processes, this signaling leads to the repression of adipocyte proliferation but accelerated differentiation (An et al. 2013). An interesting and relevant finding in studies with human heart samples reported a role for canonical Hippo signaling in arrhythmogenic cardiomyopathy (AC) in which fibroblasts are replaced by adipocytes, leading to arrhythmias and sudden cardiac death (Chen et al. 2014).

Mechanisms for the promotion of adipogenesis involved upstream activators of Mst1/2, including Merlin/NF2 and depression in levels of TEAD, with reduced expression of the TEAD gene program. Mst1/2 has also been identified as a critical mediator of homeostasis of the physical state, mass, and function of pancreatic β‐cells in vivo and in vitro. Inhibition of Mst1/2 phosphorylation by inhibitors IHMT‐MST1‐39 and ‐19 in diabetic mouse models demonstrated protection of β cells and associated depressions in levels of fasting glucose and improvements in insulin resistance (Wang, Qi, et al. 2023; Wu et al. 2022). IHMT‐MST1‐39 also activated the pathway controlled by AMPK (Wang, Qi, et al. 2023), which is a target of the anti‐diabetic drug metformin.

Excellent reviews have summarized the role of metabolic regulation engaging Hippo signaling at the level of organs and cells (Ardestani et al. 2018; Ardestani and Maedler 2018) with emphasis on its significance for metabolic homeostasis and in disease states such as cardiovascular disorders, obesity, and T2D. A model proposed for this regulation includes the regulation of Hippo by YAP activation via suppression of AMPK activity when cells are faced with high energy levels, promotion of glycolysis, and enhanced mitochondrial oxidative phosphorylation. Other mechanisms proposed are the sensing of the nutrient environment, which when low activates AMPK thereby either directly phosphorylating YAP or LATS. Also proposed are post‐translational modifications of the RHO by prenylation or YAP by O‐GlcNAcylation (O‐linked b‐*N*‐acetylglucosamine transferase [OGT]).

It is also relevant that the YAP modifications via different mechanisms can induce metabolism‐dependent regulatory mechanisms of the Hippo pathway (Ardestani et al. 2018). As an example, one mode of this regulation is the activation of AMP‐activated kinase, when suppressed by glucose signaling activates YAP. Moreover, in conditions of low nutrition, active AMPK directly phosphorylates YAP and indirectly activates LATS1/2. As reviewed by Ardestani et al. (2018) this control mechanism is present and important in homeostasis and metabolic disorders in metabolically active tissues apart from liver, including pancreatic β‐cells, adipose tissue, and the heart, all of which are regulated by Pak1.

Our goal in the above section of the review was to highlight a role for Pak1 and Hippo signaling acting in concert with homeostasis of the myocardium, adipose tissue, and pancreatic β‐cells. In summary, evidence presented above indicates that Pak1 and Hippo signaling do share a common role in the homeostasis of multiple tissues in adults. Ablation of Pak1 leads to a susceptibility to stresses faced by the myocardium, adipose tissue, and β‐cells. Ablation or downregulation of upstream elements controlling YAP/TAZ nuclear translocation also increases the stress sensitivity of these tissues. Although there are indications that these two cascades may act in concert in homeostasis and are vulnerable in pathological processes, evidence supports this regulatory mechanism is fragmented and in need of experimental approaches explicitly testing this hypothesis. An important consideration in our discussion is the targeting of PAK1/Hippo in therapeutic approaches in cancer therapy, often without discussion of off‐target effects inducing dysfunction in the heart, adipose tissue, and β‐cells. Our hope is that our discussion will stimulate this research.

## 7 Therapeutic Approaches to Control of Pak1 Activity

Evidence indicating that activation of Pak1 may be of potential therapeutic benefit in dysfunctional stress responses resulting in cardiac remodeling, mechanics, and electrophysiology stimulated our laboratory to investigate the possibility of activating Pak1. Moreover, our findings of the effects of alterations in adipose tissue in Pak^−/−^ mice also provided support for this effort. Additional support for developing ways of activating Pak1 therapeutically is in the recent report that enrichment of Pak1 specifically in β‐cells promotes insulin biogenesis and reduces cell death, resulting in suppression of diet‐induced glucose intolerance (Ahn et al. 2024). However, this therapeutic approach must be carried out with knowledge of evidence that increased Pak1 activity plays a role in adverse aging mechanisms, neurodegenerative disorders, and oncogenesis and metastasis in various cancers (Sankaran et al. 2023). In view of the evidence that Pak1 promotes tumorigenesis, there is an extensive literature on the potential of inhibition of Pak1 in cancer therapy (Eswaran et al. 2012). A related topic relevant to the present review is the investigation by Dammann et al. (2018) on whether inhibition of Pak1 activity may have application in chemoprevention of cancer in patients with T2D. Based on correlations garnered from the STRING databases, these investigators identified signaling cascades involving Pak1‐binding partners in T2D and the effects of anti‐diabetic drugs on these cascades. They speculated that the insulin response enhancers, thiazolidinediones (PPAR‐gamma agonists) may be important to test in clinical trials.

### 7.1 Activation of Pak1 via Targeting Sphingosine‐1‐Phosphate Receptors (S1PR)

There is evidence that activation of Pak1 signaling can be promoted by engagement of sphingosine‐related control processes. Bioactive lipid molecules related to sphingosine play important roles in diverse regulation across many organs. The kinase, sphingosine kinase 1, phosphorylates sphingosine, resulting in the generation of a potent mediator, sphingosine‐1‐phosphate (S1P), which binds to membrane receptors (S1PR) expressed in most human cell types. This signaling pathway has diverse functions in many diseases such as adverse lymphocyte trafficking in multiple sclerosis, cognitive decline in diabetes, various neurological disorders, diseases in cardiac electrical/mechanical activity and remodeling, as well as vascular and bronchial dysfunctions (Bascuñana et al. 2020; Bravo et al. 2022; Foran et al. 2024; He et al. 2023; Li et al. 2024; McGinley and Cohen 2021; Wang, Zhang, et al. 2024). A recent review summarized the protective role of S1PR agonists in ischemia reperfusion injury with application to multiple organ systems including heart, lungs, brain, liver, and intestines (Wang, Zhang, et al. 2024). However, there was no discussion of the role of this signaling path in adipose tissue (Wang, Zhang, et al. 2024). Notably, the main target for investigations of therapeutic applications has been recurrent and active progressive multiple sclerosis (MS). These studies produced a wealth of evidence that has identified the immunomodulatory agent fingolimod, also known as gilenya and referred to as FTY720. FTY720 modulates S1PR, and like sphingosine, is phosphorylated to FTY720‐P by sphingosine kinase. FTY720‐P binds to the S1PR and then induces inhibition of S1P signaling (Chun and Hartung 2010). Early clues in the development of FTY720 were studies reported by Bokoch et al. (1998), who demonstrated activation of Pak1 by sphingosine and several related lipids but not by ceramide. As FTY720 is a sphingosine analog, this observation suggests that FTY720 may similarly activate Pak1. These studies were followed by the identification of S1P, a previously undiscovered metabolite of sphingosine (Zhang et al. 1991). At the same time, there was a report that the mechanism of action of FTY720, then an emerging immunosuppressive agent, involved the inhibition of lymphocyte trafficking from lymph nodes via modulation of the S1PR (Yanagawa et al. 1998). FTY720 was approved in 2010 as an immunosuppressant for relapsing forms of MS with demonstration of improvements in the relapse rate, disability progress, and other endpoints (Kappos et al. 2010). A transient adverse effect was bradycardia, which is relevant to our discussion of the role of FTY720 in cardiac arrhythmias. Moreover, FTY720 also functions as an upstream activator of PP2A (Hirata et al. 2021; Rahman et al. 2016; Shrestha et al. 2018; Velmurugan et al. 2018; Vicente et al. 2020), a well‐characterized serine/threonine phosphatase involved in multiple cellular processes. This regulatory link further supports the hypothesis that FTY720 enhances Pak1‐mediated signaling and provides a mechanistic explanation for their shared functional roles in adipocytes, particularly in modulating Akt dephosphorylation. The convergence of these pathways suggests that FTY720 may exert broader effects on cellular metabolism through coordinated regulation of Pak1 and PP2A activity, warranting further investigation.

Apart from FTY720, there are three other currently available S1PR receptor modulators developed for the treatment of MS. These include Siponimod (Mayzent), Ozanimod (Xeposia), and Ponesimod (Ponvory) (Nie and Syed 2024). New agents are orally active. Analysis of the clinical use of these agents indicated that outcomes with the various agents were similar, but preferences for use by clinicians are based on the ease of clinical management with the various agents (Keenan et al. 2024). An interesting aspect of the availability of these newer agents is whether their effectiveness in diseases other than MS is different. Little evidence is available for this possibility. The second‐generation modulator of SIPR, siponimod or Mayzent, was approved for clinical use in progressive MS. Short‐term treatment of patients with siponimod has been reported to mitigate adverse effects of MS on autonomic function, in which there is a preponderance of vagal dominance. These data indicate that there is an impact on cardiovascular function that may be different from FTY720. However, with longer term treatment, there is reversion of autonomic control restoring adrenergic regulation, which also occurs in FTY720‐treated patients (Constantinescu et al. 2024).

### 7.2 Protective Effects of FTY720 in the Stressed Myocardium

A finding leading to our investigations of protective effects of FTY720 in the stressed myocardium was evidence that deletion of the gene encoding sphingosine kinase 1 in a mouse model induced a loss of the protection of ischemic preconditioning cells stressed by ischemia reperfusion (I/R) injury (Jin et al. 2007). We tested the effects of FTY720 on I/R injury in isolated hearts and SAN (Egom et al. 2010, 2011). Our initial set of results provided the first evidence that FTY720 suppresses I/R injury and associated arrhythmogenesis (Egom et al. 2010). We suggested in this first publication that this protective effect most likely involved activation of Pak1. In a follow‐up publication, we reported that treatment with FTY720 induced a protection in hearts stressed by hypoxic/ischemic cell injury (Egom et al. 2011). This protection did not occur in hearts of Pak1^−/−^ mice. A recent review has supported our earlier conclusions by summarizing the role of S1PR agonists, including FTY720, in the prevention and treatment of I/R injury in many organs apart from the heart (Wang, Zhang, et al. 2024). Based on our evidence, we speculated that this effect involved Pak1/Akt1 and NO signaling induced by FTY720. In view of these promising results, we proceeded to test whether Pak1 plays a role in cardiac hypertrophy induced by pressure overload and Angiotensin II treatment in a mouse model with cardiac‐specific Pak1 deletion Pak1(cko) (Liu et al. 2011, 2013). With prolonged stress, Pak1^fl/fl^ hearts demonstrated hypertrophy, whereas the Pak1(cko) hearts progressed and transitioned to heart failure. Although FTY720 treatment demonstrated a protective effect in prevention of the hypertrophic stress in the Pak1^fl/fl^ mice, there was no effect of FTY720 in the Pak1(cko) hearts (Liu et al. 2011). We then demonstrated that FTY720 is also able to reverse effects of existing pressure overload to induce hypertrophy and fibrosis in Pak1^fl/fl^ hearts, but not in Pak1(cko) hearts (Liu et al. 2013). Our evidence indicated that the mechanism of action of FTY720 involved negative control of NFAT activity and reduced expression of periostin. Investigations of AngII‐induced hypertrophic stress in modifying the expression of small‐conductance Ca^2+^‐activated K^+^(SK2) channel expression in murine hearts provide further support for the role of Pak1 in cardiac stress (Yang et al. 2022). Alterations in SK2 currents occurred with AngII treatment with modifications in action potential duration (APD). Loss of Pak1 function in Pak1(cko) hearts also induced adverse remodeling, decreased contractility, and increased SK2 expression in AngII stress. These effects were increased by silencing by shRNA‐Pak1 and ameliorated by treatment with FTY720 (Yang et al. 2022). In a more recent investigation, we tested the effects of FTY720 chronic treatment on hearts of mice harboring a variant of α‐tropomyosin (Tm) gene expressing an HCM‐inducing mutant, α‐Tm (E180G) (Ryba et al. 2019). FTY720 treatment improved the HCM‐related diastolic dysfunction, which is a significant factor in the adverse response to the elevated Ca^2+^ responsiveness of the myofilament in this model. Our evidence indicated that this increased response to Ca in this HCM model is due to oxidative modifications promoted by increased NADPH activity. With FTY720 treatment, there was a downregulation of NOX2 and an alleviation of oxidative modifications of the myofilaments, resulting in a return of the myofilament response to Ca^2+^ closer to control levels. However, fibrosis associated with the HCM was not prevented in this model. Interestingly, a recent study reported direct effects of FTY720 and some analogs as sarcomere inhibitors inducing a reduction in myofilament Ca^2+^ sensitivity (Kondacs et al. 2024).

### 7.3 Effects of FTY720 in Adipose Tissue, Obesity, and Insulin Signaling

There is substantial evidence indicative of pleiotropic effects of FTY720 supporting its therapeutic potential not only in cardiovascular dysfunction but also obesity, chronic metabolic dysfunction, insulin resistance, β‐cell dysfunction, T2D, and inflammation. Emerging evidence highlights S1PR signaling in these pathologies and offers potential therapeutic strategies for metabolic disorders. The S1P modulator FTY720 has been extensively studied for its effects on glucose metabolism, adipose tissue function, and inflammation in various preclinical models. In HFD‐induced obesity mice, Kendall et al. demonstrated that FTY720 prevented weight gain, improved insulin sensitivity, and reduced inflammation in adipose tissue (AT) (Kendall and Hupfeld 2008). By inducing lymphopenia, FTY720 decreased AT lymphocytes and macrophages and shifted macrophages from a pro‐inflammatory M1 phenotype to an anti‐inflammatory M2 phenotype. Despite equal food intake across groups, FTY720‐treated mice showed halted weight gain and improved glucose tolerance, highlighting its potential to modulate immune cell infiltration and function in obesity‐related insulin resistance. Similarly, Moon et al. revealed FTY720's dual regulatory role in adipogenesis and lipolysis (Moon et al. 2012). In vivo, FTY720 treatment in C57B/6J mice fed a HFD mitigated fat accumulation, reduced adipocyte size, and enhanced lipolysis by modulating the expression of key regulators, such as hormone‐sensitive lipase (HSL), adipose triglyceride lipase (ATGL), and perilipin. In vitro, FTY720 inhibited lipid accumulation in preadipocytes and promoted lipolysis, further reinforcing its role in regulating AT metabolism. Although it has not yet been demonstrated that FTY720 activates Pak1 in adipose tissue, these findings align with our observations in the Pak1^−/−^ model, where Pak1 deletion influenced adipose tissue by increasing the expression of key lipogenesis‐related genes, including acetyl‐CoA carboxylase 1 (ACC1), ATP‐citrate lyase (ACLY), fatty acid synthase (FASN), and fatty acid transport protein 1 (FATP1) which are essential for fatty acid synthesis and transport, driving lipogenesis (Munoz et al. 2024). Additionally, we observed increased Akt phosphorylation in the absence of Pak1, suggesting a potential regulatory mechanism involving a Pak1‐Akt signaling axis. The absence of Pak1 appears to enhance Akt activity, promoting lipogenesis by upregulating these lipogenic genes. This suggests that Pak1 may act as a negative regulator of lipogenesis, potentially exerting inhibitory control over Akt signaling in adipose tissue. Based on this, we propose that FTY720, through the activation of Pak1, could prevent adiposity by modulating lipolysis, lipogenesis, and adipogenesis.

Beyond its effects on AT, the S1P signaling pathway has been shown to influence broader metabolic processes, including systemic inflammation and hepatic lipid metabolism. Loss of S1PR3 in HFD‐fed mice was found to worsen AT inflammation, marked by elevated pro‐inflammatory cytokines (TNF‐α, IL‐6), increased macrophage infiltration, and decreased levels of insulin‐sensitizing factors such as adiponectin and PPARγ (Chakrabarty et al. 2022). In the liver, S1PR3 deficiency led to heightened steatosis, inflammation, and increased expression of lipogenic markers (CD36, sterol regulatory element‐binding protein 1c, SREBP1c). S1PR3 signaling also promoted adipogenesis in vitro, supporting healthy AT expansion to buffer lipid overflow. In the same study, T2D patients treated with rosiglitazone exhibited elevated levels of S1P, emphasizing the significant role of S1P‐S1PR3 signaling as a critical regulator of metabolic homeostasis. Asano et al. (2023) explored the contrasting roles of S1PR1 and S1PR2 in *ob/ob* mice. They showed that activating S1PR1 with an agonist (SEW‐2871) reduced body and fat weight, improved glucose tolerance, and suppressed adipocyte inflammation by promoting anti‐inflammatory M2 macrophages while reducing pro‐inflammatory M1 macrophages in AT. Conversely, blocking S1PR2 with an antagonist (JTE‐013) yielded similar metabolic benefits, including reduced adipocyte hypertrophy and improved insulin sensitivity. These studies collectively underline the complexity of S1P receptor signaling and its potential as a therapeutic target for metabolic diseases. In a nonhuman primate (NHP) model of spontaneousT2D, Wang et al. showed that FTY720 improved glycemic control, β‐cell function, and insulin sensitivity, while also reducing nephropathy and improving cardiac function (Wang, Liu, et al. 2018). Mechanistically, FTY720 modulated immune cell profiles, decreasing pro‐inflammatory T lymphocytes (CD4+ and CD8+) while increasing dendritic cells, which likely protected β‐cells from autoimmune destruction. In a complementary study, Zhao, Choi, et al. (2012) demonstrated that FTY720 promotes β‐cell regeneration in db/db mice through mechanisms involving PI3K‐dependent regulation of cyclin D3 and p57KIP2 to enhance β‐cell survival and proliferation while reducing apoptosis. Beyond obesity and T2D, FTY720 has also shown promise in autoimmune type 1 diabetes. In a study using the nonobese diabetic (NOD) mouse model of spontaneous type 1 diabetes, FTY720 was shown to stabilize tertiary lymphoid organs (TLOs) in the pancreas, which develop during disease progression and contribute to local immune activation and β‐cell destruction. Continuous FTY720 treatment reduced lymphocyte trafficking, preserved β‐cell compartmentalization, and effectively prevented the onset of diabetes (Penaranda et al. 2010). These findings highlight the critical role of TLO integrity in diabetes progression and suggest that FTY720's ability to stabilize these structures may serve as a promising therapeutic strategy. Taken together, these studies highlight FTY720's multifaceted role in metabolic regulation, spanning immune modulation, adipose tissue metabolism, β‐cell preservation, and systemic inflammation. Interestingly, studies of the progression of cognitive decline in a diabetic mouse model with induction of diabetes by HFD and STZ administration demonstrated that in vivo treatment with FTY720 appears to be due to neuroprotective effects (Li et al. 2024). The FTY720 treatment enhanced hippocampal autophagy and inhibited apoptosis via the AMPK/mTOR signaling pathway.

### 7.4 Bioactive Peptides and Activation of Pak1

A therapeutic approach to the activation of Pak1 that we have tested is the development of a bioactive peptide. The Pak1 activation peptide, PAP, was derived from the autoinhibitory domain (TSNSQKYMSFTDKSA) and linked to the HIV‐1 trans‐activating regulatory protein (Wang, Wang, et al. 2014). Using both in vitro and in vivo models, we tested the effect of PAP on Ang II‐induced hypertrophic and arrhythmic responses. In cultured neonatal ventricular myocytes, PAP increased Pak1 phosphorylation and enzyme activity and reduced Ang II‐induced hypertrophy. PAP administration also reduced the ANG II hypertrophy in mice. PAP also antagonized both the trigger and the substrate for ventricular arrhythmias associated with Ang II stress. These studies established the first step in developing small molecule peptidomimetics for Pak1 activation.

## 8 Future Directions

Our analysis of the interactive signaling among the myocardium, adipose tissue, and pancreatic β‐cells indicates future directions and needs related to the role of Pak1 kinase activity in these paths of communication.

There is a need for the determination of the broad effects of stimulation of Pak1 kinase activity as determined by multi‐omic and biomarker investigations in animals and humans with metabolic syndrome. In turn, there is a need for the determination of the broad effects of inhibition of Pak1 kinase activity on cardiac function, adipose, and pancreatic function in humans and animal models with cancer. The use of an artificial intelligence (AI) platform should be considered as a strategic approach to sort out the complex effects of Pak1 in this context. These findings can then proceed to validation and establishment of priorities for research.

There is a lack of understanding and a requirement for in‐depth investigations of the role of Pak1 in the control of the state and function of adipose tissue and its participation in signaling.

Evidence that the immuno‐modulator agent FTY720 is effective in activating Pak1 in a context‐dependent manner needs to be followed up with further understanding of mechanisms and further development of similar compounds that are safe and more effective.

Continued molecularly oriented research is needed with the aim of developing specific approaches for the control of Pak1 kinase activity and scaffolding activity. The application of up‐to‐date approaches such as “alpha fold” with validation and a path toward the development of mechanisms by approaches for control by site‐specific phosphorylation, peptides, or small molecules is needed.

A major issue is the need for identification, clarification, and understanding of the intra‐ and extra‐cellular signaling determining the ON/OFF states of Hippo signaling and their roles in therapy for metabolic syndrome and cancers.

There is a lack of understanding and much to be learned about the role of Pak1 in canonical and non‐canonical Hippo signaling, with emphasis on its regulatory role in specific cell types. There is a need for more consideration of overlapping regulatory mechanisms in Hippo signaling among various organs. This understanding is essential to the use of inhibitors and activators of Hippo signaling in treatments related to cancer and non‐cancer pathologies. In general, there is a need to test whether the promotion of YAP/TAZ signaling to the nucleus is able to ameliorate the pathological course of metabolic syndrome.

Although a comprehensive discussion of other Pak isoforms is beyond the scope of this review, there is important and salient evidence related to our considerations of Pak2 and in need of further research. This evidence has demonstrated a significant role of Pak2 in cardiac regeneration, stress signaling, and mechano‐transduction. Peng et al. (2016) reported that the activation of Pak2 downstream of Cdc42 and Rac‐1 mediates heart regeneration in zebrafish. There is also evidence that Pak2, like Pak1, plays a role in mechano‐transduction, where it shows protection from cell death and promotion of cell survival (Campbell et al. 2019). Binder et al. (2022) reported that deletion of Pak2 resulted in altered proteostasis of NRF2 (nuclear factor erythroid 2‐related factor 2) reducing its antioxidant activity and leading to stress. Binder et al. (2022) also report a decrease in the expression of Pak2 with an increase in NRF2 in human myocardium isolated from patients with dilated dysfunction. Moreover, Zhang et al. (2025) reported the protective effects of Pak2 expression in HFpEF by suppressing ER stress. A finding relevant to our review is the evidence that Pak2 is an important element in a signaling cascade leading to an effect of interferon gamma‐inducible protein 10 (CXCL10) to induce decreased viability of β‐cells (Schulthess et al. 2009). CXLC10 treatment of β‐cells suppressed insulin secretion and reduced the abundance of β‐cells in association with a cleavage of Pak2 that modified Akt signaling to promote apoptosis.

We have previously considered interactions between the Notch and Hippo signaling pathways. There is also evidence that Notch activation requires Pak1 under specific regulatory pathways (Vadlamudi et al. 2005). Moreover, there has been a report of the binding of the Notch intracellular domain directly to Pak1, altering its location (Yoon et al. 2016). Thus, future experiments should consider the influence of both Notch and Hippo signaling regarding proposed therapeutic interventions affecting Pak1 activity. Ultimately, future research should pave the way for innovative therapeutic strategies targeting Pak1, offering hope for improved management and treatment of complex metabolic and cardiovascular disorders.

## Author Contributions

Authors contributed equally to the conceptualization, writing, and development of the paper.

## Conflicts of Interest

The authors declare no conflicts of interest.

## Data Availability

The authors have nothing to report.
